# *dlx* and *sp6-9* Control Optic Cup Regeneration in a Prototypic Eye

**DOI:** 10.1371/journal.pgen.1002226

**Published:** 2011-08-11

**Authors:** Sylvain W. Lapan, Peter W. Reddien

**Affiliations:** 1 Whitehead Institute for Biomedical Research, Cambridge, Massachusetts, United States of America; 2 Department of Biology, Massachusetts Institute of Technology, Cambridge, Massachusetts, United States of America; 3 Howard Hughes Medical Institute, Chevy Chase, Maryland, United States of America; New York University, United States of America

## Abstract

Optic cups are a structural feature of diverse eyes, from simple pit eyes to camera eyes of vertebrates and cephalopods. We used the planarian prototypic eye as a model to study the genetic control of optic cup formation and regeneration. We identified two genes encoding transcription factors, *sp6-9* and *dlx*, that were expressed in the eye specifically in the optic cup and not the photoreceptor neurons. RNAi of these genes prevented formation of visible optic cups during regeneration. Planarian regeneration requires an adult proliferative cell population with stem cell-like properties called the neoblasts. We found that optic cup formation occurred only after migration of progressively differentiating progenitor cells from the neoblast population. The eye regeneration defect caused by *dlx* and *sp6-9* RNAi can be explained by a failure to generate these early optic cup progenitors. Dlx and Sp6-9 genes function as a module during the development of diverse animal appendages, including vertebrate and insect limbs. Our work reveals a novel function for this gene pair in the development of a fundamental eye component, and it utilizes these genes to demonstrate a mechanism for total organ regeneration in which extensive cell movement separates new cell specification from organ morphogenesis.

## Author Summary

Some invertebrates, such as planarians and *Hydra*, can regenerate fully after amputations that remove large parts of the body. We investigated how cells in the body of planarians provide new cells for eye regeneration after complete head removal. Planarians possess highly potent regenerative cells (neoblasts) in a compartment inside the worm, and these cells must be present in a body fragment for it to regenerate. We identify a pair of transcription factors, *sp6-9* and *dlx*, that are expressed in the optic cup, and use expression of these genes as markers to demonstrate that lineage restriction of eye cells during regeneration begins within the neoblast compartment. *dlx* and *sp6-9* are essential for formation of optic cup progenitors, and inhibition of these genes with RNA interference results in eyes that lack optic cups after regeneration. During eye development in both flies and vertebrates, progenitors form within a patterned epithelium. Interestingly, planarian eye precursors only aggregate once they have stopped cycling and undergone extensive migration. At this stage they already express markers of the terminally differentiated state. Therefore, we identify a mechanism for eye formation during regeneration and a novel function for a conserved gene pair in eye regeneration.

## Introduction

Animal retinas are susceptible to damage and degeneration from injury and because of sensitivity to light. Multiple vertebrates have evolved the ability to regenerate ocular tissue following damage or degeneration. In zebrafish, proliferating marginal zone cells, specialized rod progenitors, and Müller glia reside within the retina and are sources of regenerative tissue [1]. In urodele amphibians, cells of the retinal pigment epithelium can act as a source of new retinal neurons in the adult [2]. Some invertebrates, such as planarians, are also capable of eye regeneration. Unlike vertebrates, planarians can regenerate eyes completely *de novo*, using a population of cells that resides entirely outside of the eye.

**Figure 1:**
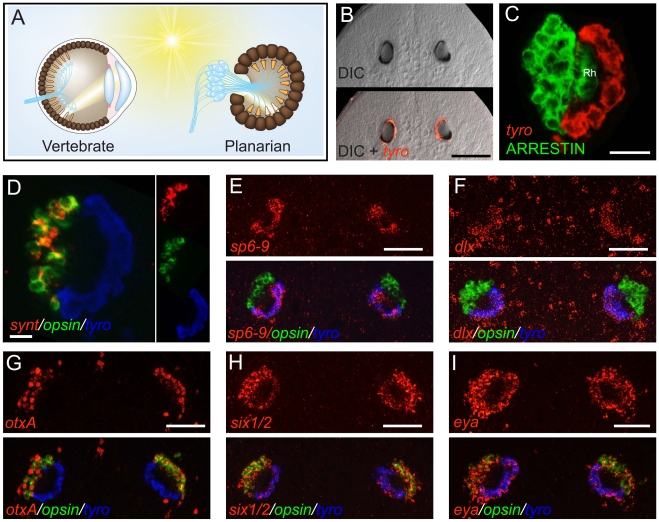
The planarian optic pigment cup expresses *dlx* and *sp6-9*. Anterior is up in all images, and all eyes are shown at day 6 of regeneration except in (B). Fluorescent images are fluorescent *in situ* hybridizations (FISH) unless otherwise noted. (A) Schematic highlighting similarities between the vertebrate (left) and planarian (right) optic cup. Light-sensing organelles (orange), neuronal cell bodies and processes (blue), and pigmented cells of the optic cup (brown) are depicted. Neural circuitry of the vertebrate retina is highly simplified. (B) *Smed*-*tyrosinase* (*tyro*) is expressed in the optic (pigment) cup of an intact planarian. (C) Planarian photoreceptor neurons, labeled by an anti-ARRESTIN antibody [60] are adjacent to the pigment cup and extend rhabdomeres (Rh) into the cup lumen. (D) The optic cup does not express *opsin* or *synaptotagmin (synt)*. (E–I) Expression of transcription factors in the planarian eye during head regeneration. *sp6-9* (E) and *dlx* (F) are expressed in the optic cup. (G) *otxA* is expressed in the photoreceptor neurons, similar to the case for *D. japonica* [20]. *six1/2* (H) and *eya* (I) are expressed in both the optic cup and photoreceptor neurons, similar to the case for other planarian species [3]–[4]. Scale bars, 100 µm (B, D), 50 µm (C, E–I).

The eyes of planarians are substantially simpler than vertebrate camera eyes, but there are important similarities between the two structures nonetheless. Eye formation in planarians, vertebrates, and other animals involves common genes such as *sine oculis* and *eyes absent* [3]–[4]. Furthermore, in both vertebrates and planarians, specialized pigment cells are organized such that they directly abut photoreceptive organelles in an optic cup. In vertebrates, cells of the retinal pigment epithelium (RPE) contact the outer segments of photoreceptor neurons from an adjacent layer of the optic cup. In planarians, the optic cup is entirely formed of pigment cells (it is commonly termed the “pigment cup”) and photoreceptor neurons project rhabdomeres into the cup [5] (Figure 1A and Video S1). A primary function of pigmented optic cups in simple eyes is to absorb incoming light prior to detection by photoreceptors [6], as this creates shade that allows the eye and brain to resolve the direction of incoming light. Light absorption is also an important function of the vertebrate RPE [7], although vertebrate eyes use sophisticated image-forming mechanisms for vision with spatial resolution.

Planarian eyes can regenerate even after decapitation, from tissue that originally resided far from the head. Planarian regeneration is possible because of a population of proliferative cells, the neoblasts, that includes pluripotent stem cells [8] as well as all cycling somatic cells of the adult. The neoblasts are distributed throughout the body in the parenchyma of the adult [9], and new tissue is produced at wounds by localized increase in neoblast proliferation, followed by cell cycle exit and differentiation [10]. Wounding in areas devoid of neoblasts ultimately results in localization of cycling cells at the wound site, indicating that cell migration can be important for repair of at least some injuries [10]. Furthermore, immediate neoblast descendants are more peripherally located than the neoblasts, suggesting that cell movements occur during differentiation [11]–[12]. In prior studies, neoblasts and their descendants were examined *in vivo* as large populations of cells with unidentified lineage and fate. Therefore, very little is currently known about the cellular and genetic events that occur between the pluripotent state and the terminally differentiated state during regeneration of specific organs such as the eye.

Here we identify the conserved transcription factors *dlx* and *sp6-9* as novel regulators of planarian eye regeneration. These genes are expressed at early stages of pigment cup progenitor specification and are required for regeneration of the cup. We find that progenitors of pigment cup cells and photoreceptor neurons form distinct mesenchymal populations substantially before differentiation and morphogenesis. Our genetic characterization of the pigment cup allows us to identify lineage-specified pigment cup cells within the neoblast population, at surprisingly large distances from the final position of the regenerating eye, and we demonstrate that *sp6-9^+^/dlx^+^* eye precursors differentiate in a spatially graded manner through the blastema prior to reaching the eye. Therefore, in contrast to epithelium-based modes of eye development, planarian eye regeneration relies on a dramatic spatial decoupling of progenitor specification and morphogenesis.

## Results

### The optic (pigment) cup is defined by expression of *tyrosinase* and the transcription factors *sp6-9* and *dlx*

As in vertebrates, but unlike most protostomes, the planarian optic shading pigment is primarily composed of melanin [13]. Consistent with this, we found that the *Schmidtea mediterranea* gene *Smed-tyrosinase* (Figure S1), homologs of which are required for melanin synthesis [14], was expressed exclusively in the pigment cup region of the planarian eye (Figure 1B and 1C and Figure S2). Whereas photosensing neurons in planarians express *Smed-opsin* [15], pigment cells did not have detectable expression of *Smed-opsin*, nor the pan-neuronal markers *Smed*-*synapsin* and *Smed*-*synaptotagmin* (Figure 1D and Figure S3), indicating that pigment cup cells do not function directly in light detection and phototransduction.

Eye development is controlled by similar transcription factors in diverse animals. We sought factors that might control formation of the pigmented optic cup in planarians during regeneration by broadly screening expression patterns of conserved transcription factor-encoding genes. We identified two genes, *Smed-sp6-9* and *Smed-dlx*, that were both expressed in the regenerating eye, specifically in the optic cup of the regenerating eye (Figure 1E and 1F). These genes were also expressed in cells outside of the eye (Figure S2 and S4), including in neurons of the brain and cells of the head rim and pharynx.

*Smed-sp6-9* (*sp6-9*) clustered within the Sp6-9 zinc finger gene family (Figure S1), which diverged from the Sp1-4 and Sp5 families prior to the evolution of the Bilateria [16], suggesting *Smed-sp6-9* is equally related to vertebrate Sp6, Sp7, Sp8, and Sp9 genes. *Smed-dlx* (*dlx*) is homologous to the Distal-less family of homeobox genes (Figure S1), which have broadly important roles in development [17]. *otxA*, *sine oculis*, and *eyes absent* genes, homologs of which encode transcriptional regulatory proteins required for the development of diverse animal eyes [18]–[19], are expressed in the eyes of planarians [3]–[4], [20]. We cloned *S. mediterranea* orthologs of these genes. In contrast to *sp6-9* and *dlx*, *Smed*-*otxA* (*otxA*) was expressed specifically in the photoreceptor neurons of the eye, but not the pigment cup (Figure 1G and Figure S2) [20]. *Smed*-*eyes absent (eya)* and *Smed*-*sine oculis1/2 (six1/2)* were expressed in both photoreceptor neurons and pigment cups (Figure 1H, 1I and Figure S2) [3]–[4]. We also identified an ortholog of *dachshund* (Figure S5A), a gene with important regulatory functions in *Drosophila* eye development [21], but did not detect *Smed-dachshund* expression in the regenerating eye (Figure S5B).

Expression of transcription factors was only weakly detected in the pigment cups of intact (non-regenerating) animals (Figure S2). As the pigment of the optic cup could obscure signal in intact eyes, we used RNAi of *tyrosinase* (see below) to reduce cup pigmentation prior to fixation of animals for FISH. Even with reduced pigmentation, however, expression of *dlx*, *sp6-9*, *six-1/2* and *eya* was faintly detected in the intact optic cup (Figure S6). In summary, we have characterized eye-expressed transcription factors in planarians on the basis of expression in photoreceptor neurons and/or the optic cup during regeneration, and have identified two genes that are expressed specifically in the optic cup during regeneration.

### *dlx* and *sp6-9* are required for regeneration of pigment cups

**Figure 2:**
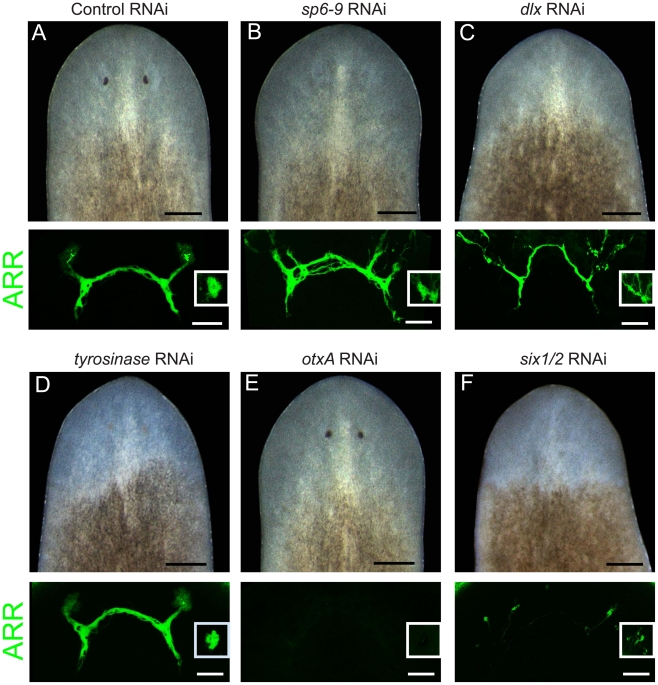
*dlx* and *sp6-9* are required for optic cup regeneration. Pigment cups are visualized in live animals on day 7 of regeneration after decapitation. Photoreceptor neurons are visualized with anti-ARRESTIN (ARR) antibody. Insets in ARR panels show a photoreceptor from the dorsal side. Penetrance in (A) is n = 10/10 (presence of pigment cups) and n = 6/6 (photoreceptor neurons that contact brain); in (B) n = 0/10 and n = 9/9; (C) n = 0/10 and 9/9; (D) n = 0/7 and n = 7/7; (E) n = 10/10 and n = 0/8; (F) n = 0/10 and n = 3/11. Scale bars, 100 µm for fluorescent images, 200 µm for live worm images.

Dlx and Sp6-9 genes have essential roles during development of several animal tissues. We therefore examined whether *dlx* and *sp6-9* loss of function could affect formation of optic cups during regeneration. RNAi of *dlx* or *sp6-9* followed by decapitation resulted in planarians that did not regenerate visible optic cups, but did make photoreceptor neurons that contacted the brain (Figure 2A–2C). Abnormal targeting of photoreceptor neuron processes in *dlx(RNAi)* and *sp6-9(RNAi)* animals was observed, possibly due to the physical absence of a cup, which typically encloses many processes (Figure 1C). *tyrosinase(RNAi)* animals had weakly pigmented optic cups, and displayed normal photoreceptor neuron morphology (Figure 2D). In contrast, RNAi of *otxA* resulted in animals that successfully made optic cups, but lacked photoreceptor neurons (Figure 2E). Consistent with *six-1/2* expression in both pigment cups and photoreceptor neurons, RNAi of this gene strongly affected pigment cup regeneration and photoreceptor neuron formation (Figure 2F), as previously observed [3]. We were unable to observe an eye defect following RNAi of *dachshund* (n = 8/8 animals regenerated pigment cups).

*dlx* and *sp6-9* were also required for eye regeneration following targeted removal of the eye without decapitation. Control animals regenerated a small pigment cup within 7 days of surgical excision of the eye. However, *dlx(RNAi)* and *sp6-9(RNAi)* animals did not regenerate pigment cups following this type of injury (Figure S7A). In order to examine whether *dlx* and *sp6-9* are required for homeostatic maintenance of the eye in uninjured animals, dsRNA was delivered by feeding over 3–7 weeks without injury (Figure S7B). *dlx(RNAi)* animals developed lesions in the region of the eye (n = 5/9). Lesions also occurred in other areas of the body, including the tail and pre-pharyngeal region, and animals ultimately died from lysis. Homeostatic *sp6-9(RNAi)* animals exhibited gradual loss in size and pigmentation of the optic cup (n = 20/20), culminating in loss of visible optic cups in some animals (n = 4/20) after 7 weeks of RNAi (Figure S7B). Our expression and functional analyses indicate that *dlx* and *sp6-9* have essential roles in pigment cup maintenance as well as regeneration following diverse injuries to the eye.

### A population of cells directly posterior to the optic cup expresses markers of the optic cup

**Figure 3:**
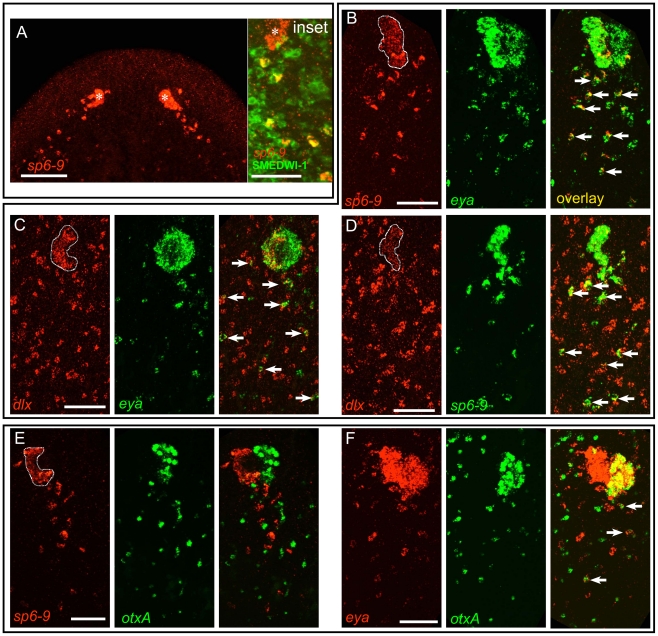
A population of mesenchymal cells posterior to the eye during regeneration expresses optic cup transcription factors. Anterior is up in all images, and all eyes and trails are in blastemas at day 6 of regeneration following decapitation. In all high magnification images of trails only one of two eyes is shown, and images are rotated slightly so that the trail fits into a vertical frame. Fluorescent images are FISH. (A) During regeneration *sp6-9* is expressed in the pigment cups (Pcup) and in trails of cells directly behind the pigment cups on the dorsal side of the animal. Inset: *sp6-9+* trail cells, but not the eye primordium, are positive for SMEDWI-1 protein. Asterisk indicates eye primordium. (B–D) Double FISH for transcription factors showing that trail cells express combinations of genes also expressed in the cup primordium. *sp6-9*-expressing (B) and *dlx*-expressing (C) cells in the Pcup and trail also express *eya*. (D) *sp6-9* and *dlx* are co-expressed in the Pcup and in many trail cells. (E) *sp6-9*-expressing cells did not express the photoreceptor neuron marker *otxA*. (F) Numerous *eya*-expressing cells did express *otxA*. Arrows show double positive cells. Pigment cup is outlined in (B–E). Scale bars, 100 µm (A), 50 µm in (B–F).

In order to understand the mechanism by which *dlx* and *sp6-9* act to promote eye regeneration, we sought to identify the source of pigment cup cells. *smedwi-1* mRNA is a marker for neoblasts, whereas SMEDWI-1 protein is a marker for neoblasts and immediate neoblast descendants, because of the fact that SMEDWI-1 protein perdures beyond expression of *smedwi-1* mRNA [10], [22]. In intact animals we were able to identify isolated cells in the dorsal anterior and in the brain that were positive for both SMEDWI-1 protein and *sp6-9* mRNA or *dlx* mRNA (Figure S8), indicating that these genes are expressed in diverse neoblast-descendant progenitor populations during homeostasis. During regeneration following decapitation, we detected a dense trail of SMEDWI-1^+^/*sp6-9^+^* cells behind the eye primordium (Figure 3A). These cells were present in a band proximal and posterior to the eye, and at the same plane of the eye on the dorso-ventral axis. This is the region imaged and analyzed in all experiments referring to a cell “trail.”

The close proximity of trail cells to the optic cup, the expression of the optic cup gene *sp6-9*, and the presence of a marker for immediate neoblast progeny cells raised the possibility that these trail cells are progenitors of optic cup cells. To explore this possibility we first sought to determine the extent of molecular similarity between trail cells and optic cup cells. Using combinatorial FISH, we found that *sp6-9^+^* trail cells co-expressed other markers of the optic cup, including *eya*, *six1/2*, and *dlx* (Figure 3B–3D and Figure S9A–S9B). The identified trail cells therefore possess an expression profile that is uniquely similar to the optic cup; outside of the eye, no other differentiated cell types, including the neighboring *sp6-9^+^* head rim cells, were found to be positive for this combination of markers (Figure S10). To test whether *sp6-9^+^* trail cells shared molecular similarity with photoreceptor neurons as well as the optic cup cells, we looked for overlap between expression of *sp6-9* and *otxA*, which is specifically expressed in the photoreceptor neurons in the eye. Cells double-positive for these markers or for *dlx*/*otxA* were observed only very rarely (Figure 3E and Figure S9C), indicating that *sp6-9^+^* trail cells share molecular identity with the pigment cup, but not with photoreceptor neurons. Numerous *otxA^+^*/*eya^+^* cells were, however, detected in mesenchymal cells posterior to the eye (Figure 3F), consistent with the fact that *eya* is also expressed in photoreceptor neurons. In summary, we identified a population of *sp6-9^+^* cells posterior to the eye during head regeneration that shares a highly similar expression profile with optic cup cells (*sp6-9^+^*/*dlx^+^*/*eya^+^*/*six1-2^+^*/*otxA^−^*), but not with any other cell type of the animal. Furthermore, we identified an adjacent population of mesenchymal blastema cells that share a similar expression profile with the photoreceptor neurons of the eye (*sp6-9^−^*/*dlx^−^*/*otxA^+^*/*eya^+^*).

**Figure 4:**
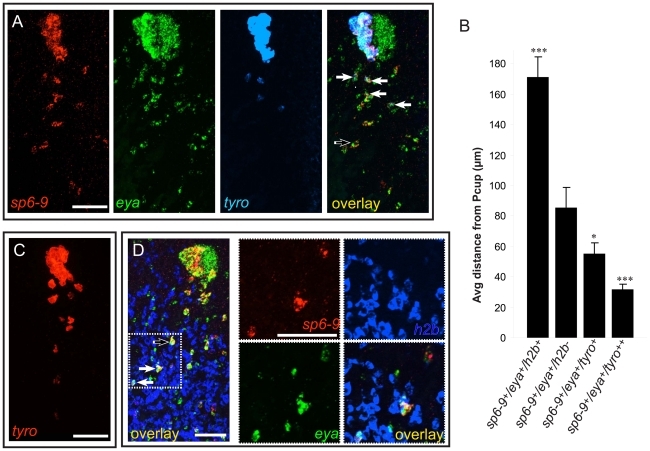
Optic cup trail cells exhibit a gradient of differentiation that correlates with distance from the cup primordium. Anterior is up in all images, and all eyes and trails are in blastemas at day 6 of regeneration following decapitation. (A) *sp6-9^+^*/*eya^+^* trail cells strongly expressing *tyrosinase* are close to the optic cup. (B) Quantification of distance of trail cells from the Pcup (mean ± s.e.m; n = 4 eyes for each category; significance by two-tailed t-test is shown relative to second bar (*sp6-9^+^*/*eya^+^*) ***P<.001, *P<.05). *tyro^++^* indicates strong, non-granular signal. (C) Weak *tyrosinase* expression can be detected in cells far from the optic cup. (D) Some *sp6-9^+^*/*eya^+^* cells express the proliferation marker *histone h2b*. Solid arrows show triple-positive cells and the open arrow shows double-positive cells. Scale bars, 50 µm.

We hypothesized that the mesenchymal trails of *sp6-9^+^*/*eya^+^*/*dlx^+^*/*six1-2^+^*/*otxA^−^* cells are progenitor cells that are the source of new pigment cup cells during eye regeneration after decapitation. Some *sp6-9^+^*/*eya^+^* cells in the trail expressed *tyrosinase* (Figure 4A). *tyrosinase* expression serves as a marker for the differentiation of optic cup cells, and its expression in the trail indicates that these cells are indeed in the process of differentiation. *sp6-9^+^*/*eya^+^*/*tyrosinase^+^* cells of the trail were typically closer to the optic cup (Figure 4A and 4B) than were *sp6-9^+^*/*eya^+^*/*tyrosinase^−^* cells. Using single rather than triple probe *in situ* hybridization conditions, some *tyrosinase^+^* cells could be detected 150 µm or more away from the pigment cup (Figure 4C). Expression of *tyrosinase* in these distant cells was consistently much weaker, further indicating that proximity to the cup primordium correlates with extent of differentiation. *tyrosinase* expression in intact animals was not detected in any cells of the body outside of the optic cup, confirming that the expression profile of the *sp6-9^+^* trail cells identified here is uniquely similar to that of optic cup cells. We refer to these cells as “optic cup trail cells,” and use detection of *sp6-9* and *eya* co-expression to identify these cells in subsequent experiments.

### Optic cup trail cells originate in the neoblast population

Differentiated cells in planarians are non-mitotic, and neoblasts are the only cycling somatic cells of the animal [9]. Surprisingly, at positions relatively far from the pigment cup, *sp6-9^+^*/*eya^+^* trail cells were observed that also expressed the proliferative marker [23] *histone h2b* (Figure 4D) and the neoblast marker [24] *smedwi-1* mRNA (Figure S11). RT-PCR analysis of FACS sorted “X1” neoblasts has indicated that the neoblast population contains heterogeneity of gene expression and possible commitment to specific lineages [25]. *smedwi-1* and *h2b* positive optic cup trail cells, described here, are the first lineage-committed neoblast subpopulation observed *in vivo*. It will be interesting to investigate whether these *sp6-9^+^*/*eya^+^* cycling cells function as stem cells, or whether they are a transient, differentiating cell type. Neither *h2b* nor *smedwi-1* expression was observed within the optic cup at any point during regeneration. Indeed, *sp6-9^+^*/*eya^+^*/*h2b^+^* cells were typically present in a region of the trail that was relatively distant from the regenerating eye primordium, in contrast to the *sp6-9^+^*/*eya^+^*/*tyrosinase^+^* cells that were closer to the eye (Figure 4B and 4D). These data indicate that there exists a distal-to-proximal distribution of progenitor trail cells with respect to the eye that begin in the dividing neoblast population (distal), exit the cell cycle and begin differentiation (intermediate), and finally differentiate fully and aggregate (proximal).

### Optic cup trail cells are a source of new eye tissue during regeneration

**Figure 5:**
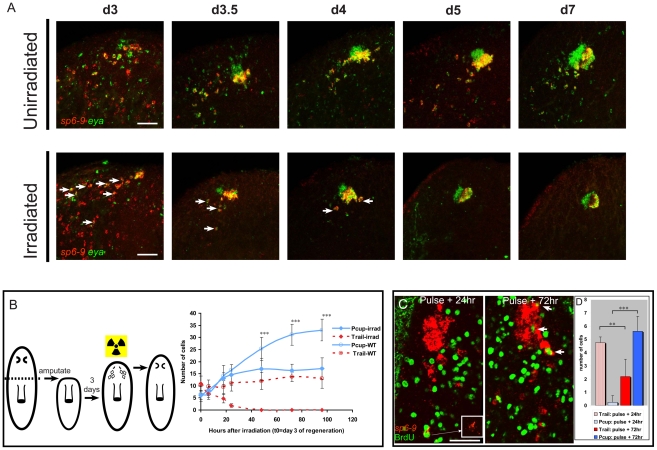
Optic cup trail cells are a source of new optic cup tissue. All fluorescent images are FISH, except for fluorescent detection of BrdU by antibody. (A) Time course of eye regeneration in unirradiated animals and animals irradiated at day 3 of regeneration following decapitation. In irradiated panels arrows indicate double positive cells. (B) Experimental design diagram and quantification of cell numbers in pigment cups (Pcups) and trails of irradiated and unirradiated animals over time (mean ± s.d; n≥9 eyes/trails; significance by two-tailed t-test is shown for the Pcup cell numbers in irradiated vs. un-irradiated ***P<.05). (C) BrdU injection at day three of regeneration after decapitation only labels trail cells after one day, but labels Pcup cells after a longer time delay. Inset: magnification of *sp6-9* signal in BrdU/*sp6-9*-positive cell. Arrowheads indicate *sp6-9^+^*/*eya^+^*/BrdU^+^ Pcup cells. (D) Quantification of BrdU^+^/*sp6-9^+^*/*eya^+^* cell numbers in the trail and Pcup 24 h and 72 h after BrdU injection (mean ± s.d; n = 5 eyes; significance by two-tailed t-test ***P<.001, **P<.005). Scale bars, 50 µm.

To further test the possibility that optic cup trail cells are the source of new optic cup tissue in regeneration, animals were irradiated with 6,000 Rads on the third day of regeneration following decapitation, a point at which aggregates of eye cells were first apparent. Irradiation permanently blocks all new cell division in planarians [9]. Nonetheless, optic cups underwent significant growth, measured in number of cells, following irradiation on day 3 of regeneration (Figure 5A and 5B). Simultaneously, the number of *sp6-9^+^*/*eya^+^* trail cells gradually decreased following irradiation. The extinction of trail cells 24–48 hours after irradiation correlated with the end of cup growth. Furthermore, the number of trail cells present shortly following irradiation approximately matched the number of cells gained by the optic cup with time following irradiation. When BrdU was injected at day three of regeneration, incorporation into *sp6-9^+^*/*eya^+^* cells was first detected distal to the cup in the trail, at 24 hours after injection (Figure 5C and 5D). Incorporation of BrdU first into distal cells of the trail provides further evidence that these cells have recently undergone S-phase or are actively in S-phase. At later time points following the BrdU pulse, however, a majority of *sp6-9^+^*/*eya^+^*/BrdU^+^ cells were located in the cup aggregate (Figure 5C and 5D). Because most cycling *sp6-9^+^*/*eya^+^* cells are located at sites distal to the eye, and no cycling cells are found in any part of the eye primordium (Figure 4D), the presence of BrdU signal in the eye three days after the BrdU pulse indicates that a net movement of *sp6-9^+^*/*eya^+^*/BrdU^+^ cells from distal to proximal to the eye occurs during regeneration. Together, these data suggest a model in which optic cup progenitors arise after decapitation and migrate toward the eye while undergoing differentiation.

### *dlx* and *sp6-9* are required for eye progenitor formation after decapitation

**Figure 6:**
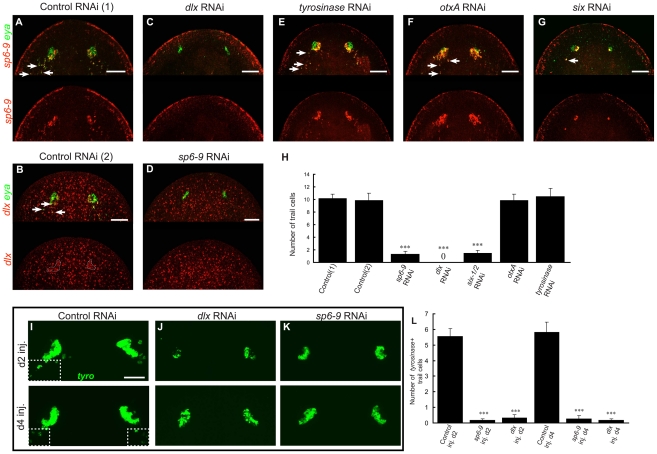
*dlx* and *sp6-9* are required for regenerative optic cup cells. All fluorescence is FISH. Day 7 regeneration blastemas are shown, anterior is up. (A–G) Photoreceptor defects following RNAi of eye-expressed genes. Control RNAi animals have co-localization of (A) *sp6-9* with *eya*, or (B) *dlx* with *eya* in the eyes and progenitor trail, n = 10/10 animals for each. (C), *dlx(RNAi)* animals do not regenerate *sp6-9^+^*/*eya^+^* cells, n = 10/10. (D) *sp6-9(RNAi)* animals do not regenerate *dlx^+^*/*eya^+^* cells, n = 10/10. (E–F) *tyrosinase(RNAi)* and *otxA(RNAi)* animals are able to regenerate *sp6-9^+^*/*eya^+^* cells. (G) *six-1/2(RNAi)* regenerate greatly reduced numbers of *sp6-9^+^*/*eya^+^* cells. (H) Quantification of the number of optic cup trail cells for RNAi conditions in (A–G). For control(2) and *sp6-9* RNAi, *dlx* and *eya* coexpression is used to count trail cells. For all other conditions *sp6-9* and *eya* coexpression is used. (I–K) RNAi of *sp6-9* and *dlx* beginning on either day 2 or day 4 of regeneration results in smaller pigment cups and fewer terminal trail cells, assessed by *tyrosinase* expression. Boxes with dashed outline in (I) enclose terminal trail cells. (L) Quantification of number of *tyrosinase* ^+^ trail cells in the indicated RNAi conditions. Arrows indicate double positive cells. Scale bars, 50 µm.

Because *sp6-9* and *dlx* are expressed in the undifferentiated cells of the optic cup trail, we sought to determine whether they are required for generation of the optic cup progenitors or for later stages of cup formation. RNAi of *dlx* to eliminate gene function resulted in animals that completely lacked *sp6-9^+^*/*eya^+^* optic cup trail cells during regeneration as well as *sp6-9^+^*/*eya^+^* double-positive signal in the optic cup primordium (Figure 6A, 6C and 6H; higher magnification images of RNAi phenotypes are in Figure S12). We obtained a similar result for *sp6-9(RNAi)* animals, which largely lacked *dlx^+^*/*eya^+^* progenitor cells (Figure 6B and 6D). *eya^+^* signal remaining in these animals reflects photoreceptor neuron expression of this gene. *tyrosinase(RNAi)* animals lacked visible eye pigment but displayed normal *sp6-9^+^*/*eya^+^* progenitor patterns (Figure 2D, Figure 6E and 6H), and eye markers were normally positioned, indicating that, unlike *sp6-9(RNAi)* and *dlx(RNAi)* animals, *tyrosinase(RNAi)* animals are able to regenerate optic cup tissue. *otxA(RNAi)* animals also had normal and abundant *sp6-9^+^*/*eya^+^* progenitors, as expected given that these animals regenerate visible pigment cups (Figure 6F and 6H). RNAi of *six1/2* led to severe reduction in numbers of both pigment cup progenitors and photoreceptor neurons following regeneration (Figure 6G and 6H). We verified the presence or absence of pigment cups in the above RNAi conditions using DIC light microscopy (Figure S13). Pigment cups (epithelial cells arranged in a crescent around a lumen) were observed in control, *tyrosinase(RNAi)*, and *otxA(RNAi)* animals; no such cellular formations were observed in *sp6-9(RNAi)*, *dlx(RNAi)*, or *six1/2(RNAi)* animals.

**Figure 7:**
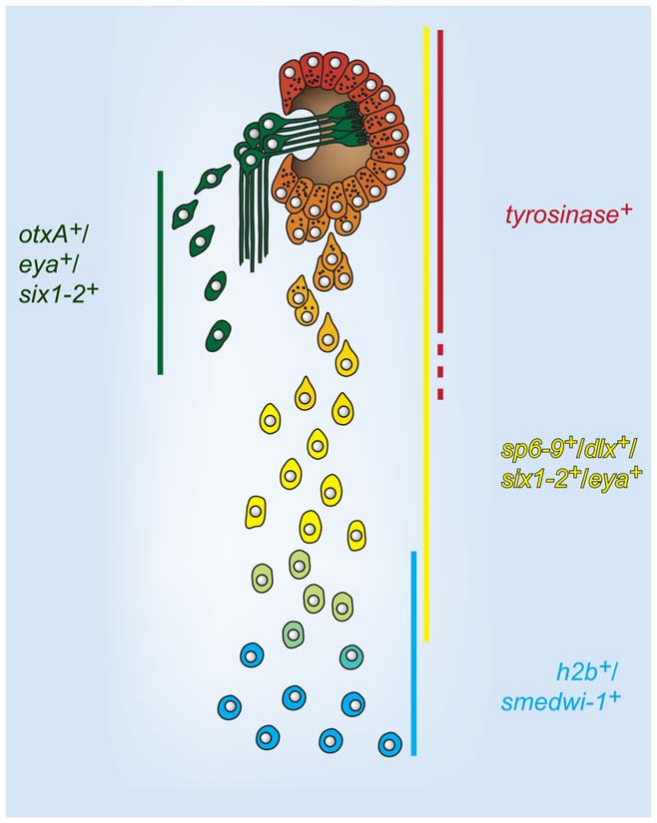
A model for cell state changes in pigment cup regeneration. Lines indicate domain of expression of the adjacent gene labels in the optic cup trail during regeneration. Regenerative photoreceptor neuron cells are shown as spatially separated for clarity. This model proposes that optic cup progenitors are specified at distance from the aggregated cells of the optic cup primordium, within the neoblast population (*h2b^+^*, *smedwi-1^+^*). Progenitors undergo changes in gene expression, including loss of neoblast markers and activation of differentiation markers, as migration towards the eye primordium proceeds. Ultimately, terminally differentiating progenitors incorporate into the eye and undergo a mesenchymal-to-epithelial transition.

The RNAi experiments described above were performed by feeding dsRNA to animals for one week prior to cutting. In order to confirm that *sp6-9* and *dlx* gene functions are required during regeneration, we applied dsRNA only after amputation by using an injection strategy. dsRNA was applied beginning at either day 2 or day 4 of regeneration following decapitation. In both cases, pigment cups were typically present at day 7 of regeneration in *dlx(RNAi)* and *sp6-9(RNAi)* animals, as expected because some progenitors are formed before the onset of RNAi (Figure 6I–6L). However, RNAi animals lacked the *tyrosinase^+^* terminal trail cells posterior to the eye (>97% reduction for *dlx* and *sp6-9* RNAi at d2) and had pigment cups that were smaller than in control animal (Figure 6I–6L and Figure S14). Therefore, inhibition of *sp6-9* and *dlx* only during regeneration successfully impaired ongoing pigment cup cell specification. Despite a reduction in total numbers, the pigment cup cells that were generated successfully aggregated into concave cups properly positioned in the head. We conclude, based on these data, that *dlx* and *sp6-9* are required for progenitor formation during regeneration of the optic cup (Figure 7).

## Discussion

### A cellular model for regeneration of the eye

All regions of the planarian body that can regenerate contain somatic dividing cells (neoblasts), which are typically identified by expression of cell cycle genes and *smedwi-1*, a piwi homolog. Recent work has shown that some of the adult dividing cells are pluripotent stem cells, called cNeoblasts, which are capable of generating all essential cells of the body [8]. Here we show that some neoblasts have gene expression and distribution that is indicative of restriction to a specific cell type, the optic cup cells of the eye. Optic cup progenitors are induced in abundance within the parenchyma of the animal after decapitation, at a distance from the (now absent) site of the lost eye as well as the aggregated primordium of the newly regenerating eye. Differentiation and migration toward the eye primordium are temporally correlated, as expression of neoblast markers is downregulated and expression of the differentiation marker *tyrosinase* is upregulated in progenitor cells approaching the eye (Figure 7). Therefore, we spatially and molecularly identify progenitors between the pluripotent state and the terminally differentiated state during regeneration of a specific tissue. The eye is an ideal system with which to address questions of how the identity of lost tissue impacts the generation of regenerative cells, as its pigmentation and restricted localization greatly simplify targeting for surgery. Therefore, many questions can now be asked about how specification, migration, and differentiation of a specific progenitor population are regulated following diverse injuries to a specific organ. For example, what is the minimal injury to the eye required for induction of eye progenitors?

Previous work has suggested that optic cup cells and photoreceptor neurons in planarians derive from common progenitors cell that expresses terminal differentiation markers of both the optic cup cells (*tryptophan hydroxylase*) and photoreceptor neurons (*arrestin*) [26]. By contrast, our work indicates that progenitors for photoreceptor neurons and cup cells exist as distinct progenitor populations prior to terminal differentiation and aggregation in the eye primordium, based on distinct expression of *dlx*/*sp6-9* and *otxA* in mesenchymal *six^+^*/*eya^+^* cells present posterior to the eye. With these results we cannot exclude that all eye cells derive from a common upstream, eye-specific progenitor. However, it is apparent that they exist as spatially distinct populations with distinct gene expression for a substantial period before differentiation and aggregation into an eye primordium. In most developing eyes, including in *Drosophila* and vertebrates, pigment cells and photoreceptor neurons are formed from a common epithelial eye field [27]. We find no epithelial eye field that contains proliferating or undifferentiated eye progenitors in the regenerating or intact planarian. Therefore, it will be interesting to determine whether the pigment cells and photoreceptor neurons likewise share a common eye-specific precursor population in this organism.

A significant consequence of development within an epithelial field is that position and numerical proportion of various cell types can be influenced by direct signaling from one cell type to another. For instance, in *Drosophila* eye development many cell fate decisions depend on direct cell-cell signaling via Notch/Delta, including the induction of primary pigment cells by adjacent cone cells [28]. Signaling between mesenchymal eye progenitors in planarians is less likely to depend on cell contact. Longer-range signaling molecules in planarians could be responsible for coordinating progenitor numbers and position. Perturbation with RNAi of Wnt [29]–[33], Bmp [34]–[36], or Fgf [37] signaling in planarians can result in ectopic eyes. It is unknown, however, whether these pathways act directly in eye progenitor specification or whether ectopic photoreceptors are the indirect result of major regional tissue changes in these RNAi animals, such as expansion of the brain. Investigation of the signaling molecules that directly act on eye progenitors might promote understanding of how progenitor numbers are coordinated during *de novo* organ regeneration.

### Transcription regulatory genes and eye regeneration

The planarian eye has important similarities to many animal eyes, including expression of regulatory transcription factors such *sine oculis* [3] and *eyes absent* [4], downstream genes such as *opsin*, *arrestin*, and *tyrosinase*, as well as the overall morphology of a pigmented cup that encapsulates photoreceptive organelles. We identify planarian *dlx* and *sp6-9* as novel regulators of optic cup progenitor formation during planarian eye regeneration. Dlx and Sp6-9 transcription factors are well conserved throughout the Eumetazoa and can function as an evolutionarily conserved module. Together, Dlx and Sp6-9 genes regulate limb outgrowth in both insects and vertebrates [38]–[42] and are important for development of the vertebrate tooth [40], [43]–[44], another appendage-like structure. Dlx and Sp6-9 family genes are also both expressed in the anterior of the body axis in *Drosophila* embryos and the basal deuterostome *Saccoglossus* [45], although a coordinated role for these two genes in the anterior of the body axis has not yet been supported by functional data. Multiple Dlx genes are expressed in the vertebrate eye from early stages of retina formation [46]–[47], and a Dlx gene is expressed in the adult *Platynereis* cup-shaped eye [48].

Planarian eye regeneration differs from eye development in a number of other animals in that it does not appear to rely on orthologs of the *pax6(eyeless)* [49] or *dachshund* genes, two important components of the *Drosophila* retinal determination gene network (RDGN). Pax6 genes are important regulators of eye development across many animal lineages [50]. *dachshund* is required for *Drosophila* eye development [51], and *dachshund* orthologs are expressed, but not functionally required, during mouse eye development [52]. Therefore, potentially ancestral eye specification genes [21] might not be involved in planarian eye regeneration. One possible explanation for this is that differences in specification mechanisms may exist between embryonic development and adult regeneration. The roles for eye regeneration genes are currently unknown in planarian embryonic eye development. Another possibility, if these genes indeed had roles in eye specification in the ancestor of Bilateria, is that planarians have diverged over evolutionary time with respect to their reliance on *pax6* and *dachshund*.

Planarians also differ from vertebrates, but are similar to *Drosophila*, in the role of Orthodenticle homologs in pigment cell specification in the eye. Vertebrate RPE development depends on *otx2* [53], and regeneration of the newt RPE is associated with expression of *otx* [54]. By contrast, in planarians the only known eye-expressed orthodenticle homolog, *otxA*, is expressed specifically in the photoreceptor neurons, and not the pigmented cells (Figure 1H) [20] and is primarily required for photoreceptor neuron specification (Figure 1N). Similarly, in *Drosophila* development, *orthodenticle* is required for photoreceptor neuron development, and a role in pigment cells of the eye has not been described [55]. Therefore, the function of Orthodenticle homologs in maintenance and specification of pigmented eye cells may differ between protostomes and deuterostomes.

Despite these differences, the demonstrated Eumetazoa-wide conservation of numerous other eye genes, as well as the demonstrated capacity of Dlx and Sp6-9 to execute deeply conserved developmental programs, indicates that investigation of this gene pair in diverse animals will be an important direction for the study of optic cup formation and regeneration. Furthermore, the identification of a highly localized eye progenitor population based on expression of *dlx* and *sp6-9* will facilitate the exploration of progenitor dynamics during regeneration.

## Materials and Methods

### Gene cloning

Genes were cloned from cDNA using gene-specific primers designed from EST databases and gene predictions (Genscan and Maker) [56]. For cDNA library generation, RNA from mixed stage regenerating animals was isolated in Trizol, and used as template for first-strand reverse transcription with Superscript III (Invitrogen). PCR using cDNA template and gene-specific primers was typically followed by a secondary, interval PCR to improve specificity and yield. The following primer sequences were used with gateway adapters or addition of T7 promoter sequence (see below) for cloning genes.

*dachshund*

5′ primer: GTGGGCTTCACACGGTTTAT

nested 5′ primer: TTGAAGAGACTAGAAATCGTTCCA

3′ primer: TTGCACAAACTTTGCAGGAG

*dlx*

5′ primer: AATGAACCTCCCACTGCA

nested 5′ primer: CAGGATCAGAAACCCAATCC

3′ primer: CGGTTATTCGAAAAATTAACTGG

*eyes absent*

5′ primer: GGCCTTTCAAAAGACGACTC

3′ primer: AAGACTCAATGCGTGGTGAA

*opsin*

5′ primer: TGGTTTCATCGGTGGTCTTT

nested 5′ primer: TGGGTTTATATCCATCAACACAAT

3′ primer: TTTTTGCACCCGTTTTCAT

*otxA*

5′ primer: CCACAAATCCCTCTCTACGG

nested 5′ primer: ACGTAGCTGGGATCAACACC

3′ primer: TGGACCTGACAAATTGTTCC

*six*

5′ primer: ATCGATATCCACGAGCCAAG

3′ primer: CCAGATTCGCATTCGTTACTT

nested 3′ primer: ACAGGACTCCGAACAAATCA

*sp6-9*

5′ primer: TTCAATAAATAACGTTGAGAGCAA

nested 5′ primer: ATCAATCTTGGCTATTGGAACG

3′ primer: TTCACAATTGTTTGTTAACGACTC

*tyrosinase*

5′ primer: TGCTCGTAATCACAATAGGCATAG

nested 5′ primer: TTTGCATCTTTCTTACCTTTGAGT

3′ primer: TTTCTTAATAGCCAAATTTCAAAGA

For gateway cloning, the following adapters were appended to the above primers

5′ primer adapter: AAGCTGGAGCTCCACCGCGG

3′ primer adapter: GGGCGAATTGGGTACCGGG

cDNA amplified by PCR was cloned into pGEM (pGEM T-Easy, Promega) for use in riboprobe and dsRNA reactions (see below). For RNAi experiments by feeding, gateway recombination was used to clone genes in pPR244, a dsRNA-expression vector, as described [24]. For determination of complete gene sequences, 5′ and 3′ RACE libaries were generated from mixed stage planarian RNA (FirstChoice RLM-RACE, Ambion). Nested primers were designed to either the 5′ or 3′ prime end of the known gene sequence, and candidate bands following the second (nested) PCR were excised, purified, cloned into pGEM, and sequenced.

### RNA synthesis

To generate template for riboprobe synthesis, amplified cDNAs were first cloned into pGEM (as above). Template was generated with same primers as were used for cloning, except that gateway adapters were not present on primers, and T7 promoter sequence was appended to the 3′ primer. Transcription reactions were performed with T7 (Promega) and either DIG-, FITC- (Roche), or DNP- (Perkin Elmer) labeled ribonucleotides. RNA was purified using ethanol precipitation with 7.5 M ammonium actetae (1∶2). Pellets were resuspended in formamide (50 µl per 25 µl synthesis reaction), and stored at −20°.

To generate template for dsRNA synthesis *in vitro*, generic primers recognizing gateway adapter sequences were used with T7 appended to either the forward or reverse primer, and PCR was performed using amplified cDNA (with gateway adapters) cloned into pGEM. T7 transcription reactions for sense and antisense RNA were performed separately, and pooled prior to phenol-chloroform extraction followed by ethanol precipitation with sodium acetate (1∶10). RNA was annealed using the following program in a thermocycler: 70°C for 10′, 37°C for 30′, 4°C for 10′.

### RNAi

RNAi was performed by feeding intact animals with *E. coli* expressing dsRNA under an inducible promoter. HT115 competent cells were transformed with pPR244 vector [57] containing the gene of interest. Negative control experiments used a 1 kb region of *unc-22*, a *Caenorhabditis elegans* gene with no significant nucleotide sequence similarity to a planarian gene. Cultures were grown in 2×YT media to an OD600 of 0.35–0.45, and then induced for two hours with 1 mM IPTG. Cultures were pelleted and resuspended in a volume of 70% liver/30% water equal to 1/300^th^ of the original volume of the culture, and stored at −80°C. Worms were fed three times prior to amputation. Amputation was performed on the day following the final feeding, and worms were fixed following one round of regeneration. For targeted eye surgery, a microsurgical blade (MSP, 15°, 3 mm depth) was inserted into the eye and the optic cup was excised upon removal of the blade. Surgery was performed one day after the third RNAi feeding, and one additional feeding was administered 4 days after surgery. For homeostasis RNAi experiments, animals were fed every four days.

For RNAi by injection, dsRNA was diluted to 4 µg/µl in water. For standard RNAi application (*dachshund* RNAi experiment), animals were amputated and injected after 30 min, and again after 24 hours. After 3 days of regeneration, animals were amputated again and injected once more after 30 min. For RNAi injection in Figure 6I–6K, animals were injected once, either on day 2 or day 4 of regeneration after decapitation. Injection was performed with a Drummond Nanoject II and 3.5″ Drummond capillaries (3-000-203-G/X). Animals were immobilized for injection with use of a peltier cooling block. Several injections of 32 nl were applied as needed to observe swelling of animals with liquid.

### Histology and imaging

Whole-mount fluorescent *in situ* hybridization (FISH) and antibody staining was performed as described [58] except that FITC-tyramide was used at 1∶500, and HRP enzyme was inactivated using 4% formaldehyde. Tyramide was generated by conjugation of succinimidyl esters of rhodamine, FITC, Cy5 and AMCA with tyramide-HCL (Roche) [59]. Riboprobes (see below) were used at 1∶800 dilution in hyb solution, except for *opsin* and *tyrosinase*, which were used at 1∶1200. Anti-ARRESTIN (VC-1) antibody (1∶5000 dilution) was kindly provided by Kiyo Agata, and SMEDWI-1 antibody (1∶1000 dilution) was obtained as described [10], [22].

For BrdU experiments, animals were injected with a solution in 5 mg/ml of BrdU (Fluka) in planarian water. Similar injection methods were used as for dsRNA injection (see above), and animals were only injected once. Animals were then fixed and labeled according to the regular FISH protocol, and following FISH development animals were treated with 2N HCl for 45 minutes at RT. BrdU was detected with rat-anti-BrdU (1∶100) antibody (Oxford Biotech) followed by incubation with anti-rat-HRP (1∶100) (Abcam) and development with commercial tyramide (Invitrogen).

Optical sectioning was performed using an Apotome, Axiocam digital camera, a Zeiss AxioImager, and Axiovision software. Images in Figure 5A, Figures S4 and S8 were generated with a Zeiss confocal microscope (LSM 700). Brightness, contrast, and gamma were adjusted as needed to improve visibility. In most images, optical sections are overlayed to show co-expression in cells across a depth of tissue. Stacks of sections were manually examined at the level of individual optical sections to determine true instances of co-expression, and instances of artifact overlapping signal created by digital overlaying were excluded from analyses.

### Phylogenetic analyses

*S. mediterranea* amino acid sequence predictions were aligned (ClustalW) with sequences of putatively homologous proteins from other metazoans. Sequences were trimmed by gblocks under the lowest stringency settings. Phylogenetic trees for *tyrosinase*, *sp6-9*, *dlx*, and *otxA* were constructed using Bayesian inference (MrBayes) with >2,000,000 generations, and >1,500 burn-in trees discarded. Branch labels display posterior probabilities.

## Supporting Information

Figure S1 Orthology of *S. mediterranea* genes. Posterior probabilities are shown on branches. See Materials and Methods for details. Dm, *Drosophila melanogaster*; Dr, *Danio rerio*; Hs, *Homo sapiens*; Gg, *Gallus gallus*; Gt, *Girardia tigrina*; Dj, *Dugesia japonica*; Nv, *Nematostella vectensis*; Sp, *Strongylocentrotus purpuratus*; Pd, *Platynereis dumerilii*; Ci, *Ciona intestinalis*; Sk, *Saccoglossus kowalevskii*; Bf, *Branchiostoma floridae*; Ce, *Caenorhabditis elegans*; Cb, *Caenorhabditis briggsae*.
(PDF)

Figure S2 Expression of eye genes in intact animals. FISH showing expression of indicated gene in whole-mount intact animals. Expression of some pigment cup-expressed genes in intact animals is partly obscured by unbleachable eye pigment. Dorsal is shown in left panel, ventral on right for each gene. Scale bars, 200 µm.
(PDF)

Figure S3 *synapsin* and *synaptotagmin* are pan-neuronally expressed and *synapsin* does not label the pigment cup. All fluorescence is FISH. Ventral view of 6 day regenerating animals showing pan-neuronal expression of (A) *synapsin* (B) *synaptotagmin* orthologs in the brain and elsewhere. As for *synaptotagmin* (Figure 1D), *synapsin* signal (C) is not detected in the optic cup. Scale bars, 100 µm.
(PDF)

Figure S4 Expression domains of *dlx* and *sp6-9* in relation to neurons of the head rim and brain. All fluorescence is FISH, animals are intact (non-regenerating), and anterior pole is facing up. (A) and (B) are dorsal views of the anterior left side of the animal, (C) and (D) are ventral views of the anterior of the animal. (A) *dlx* is expressed in diffuse cells throughout the dorsal anterior, some of which express neuronal markers. (B) *sp6-9* is prominently expressed in the head rim epidermis, as well as sparse underlying cells. These cells abut neurons but largely do not express *synaptotagmin*. (C) *dlx* is expressed in many neurons within the primary lobes of the brain as well as more lateral regions. (D) *sp6-9* is expressed in a small subset of cells located in the ventral brain, most of which express *synaptotagmin*. (E) *dlx* is also expressed in cells of the pharynx and pharynx cavity at the midbody of the animal. Pr, photoreceptors; Phx: pharynx. Scale bars, 50 µm.
(PDF)

Figure S5 *dachshund* is not detectably expressed in the regenerating eye and does not have an obvious eye phenotype following dsRNA injection. (A) Orthology of *Smed-dachshund*. Posterior probabilities are shown on branches, see Materials and Methods for details. dm, *Drosophila melanogaster*; dr, *Danio rerio*; gg, *Gallus gallus*; mm, *mus musculus*. (B) Expression analysis with FISH does not indicate expression of *Smed*-*dachshund* in the photoreceptor neurons or the optic cup in 6 day regenerating heads. *opsin* (green) and *tyrosinase* (blue) expression are used to label photoreceptor neurons and optic cup, respectively. Scale bar, 50 µm.
(PDF)

Figure S6 Expression of transcription factors in de-pigmented optic cups of intact animals. RNAi of *tyrosinase* was used to reduce melanin pigment in the optic cup. (A) DIC image of an optic cup in an animal not treated with *tyrosinase* RNAi. (B) Expression of selected transcription factors in intact eyes of *tyrosinase* RNAi animals (FISH). The crescent-shaped structure in the DIC image is the optic cup. Arrows indicate regions of the optic cup with expression of the gene labeled in the panel. Scale bar, 20 µm.
(PDF)

Figure S7 *sp6-9* and *dlx* are required for regeneration of the optic cup after excision and for homeostatic maintenance of the optic cup. (A) The optic cup was surgically removed after 3 RNAi feedings, at which point animals in all RNAi conditions appeared similar. 7 days after surgery, only control animals showed signs of optic cup regeneration. Regeneration of the optic cup was apparent in n = 10/10 control RNAi animals, n = 0/10 *dlx(RNAi)* animals and n = 0/10 *sp6-9(RNAi)* animals. (B) RNAi of *dlx* in uninjured animals resulted in worm lysis within 3 weeks. n = 5/9 worms surviving at three weeks of homeostasis had lesions in the area of the eye. RNAi of *sp6-9* in uninjured animals resulted in pigment cups that were reduced in size and pigmentation in n = 20/20 animals after 7 weeks. 4/20 of these animals lost pigment cups completely. Scale bars, 500 µm (A), 200 µm (B).
(PDF)

Figure S8 Expression of *dlx* and *sp6-9* in neoblasts or immediate neoblast descendants in intact animals. All fluorescence is FISH, animals are intact (non-regenerating). (A) and (B) are dorsal views, (C) and (D) are ventral views. (A) *dlx*-expressing cells on the dorsal side of the animal that also express SMEDWI-1 protein can be found both posterior and anterior to the photoreceptors. (B) A small population of *sp6-9*-expressing cells in the pre-pharyngeal region posterior to the photoreceptors also expresses SMEDWI-1 protein. These may represent optic cup progenitors that function during homeostasis. (C) Some *dlx*-expressing cells at the periphery of the brain lobes are positive for SMEDWI-1. (D) Most *sp6-9* positive neurons (located in a ventral region of the brain) are fully differentiated, but some cells in the anterior of this domain express SMEDWI-1. Scale bars, 50 µm.
(PDF)

Figure S9 Additional image data for Figure 3. Anterior is up in all images, and all eyes and trails are in anterior blastemas at day 6 of regeneration following decapitation. Fluorescent images are FISH. (A) *six1/2* expression and *eya* expression overlap fully in the eye aggregate and in the trail. (B) *six1/2* expression and *sp6-9* expression overlap partly in the eye aggregate and partly in the trail. (C) *dlx*-expressing cells do not detectably express *otxA*. Arrowheads indicate double-positive cells. Scale bars, 100 µm.
(PDF)

Figure S10 Overlapping expression of transcription factor combinations is not found outside of the eye region. (A) FISH showing expression of indicated genes in whole mount intact animals. Both rows show overlayed optical sections imaged from the dorsal surface. The second row is imaged at a more ventral level than the first. Arrows indicate regions with double positive cells. In intact animals, *sp6-9*, *dlx*, and *eya* expression is difficult to detect in intact pigment cups, but is apparent in new cells that are incorporating as part of homeostatic maintenance. (B) Overlayed optical sections taken from ventral surface of the animal showing that *sp6-9^+^* nerve cord cells do not detectably express *eya*, unlike optic cup cells. Scale bar, 50 µm.
(PDF)

Figure S11 Some *sp6-9^+^/eya^+^* trail cells also express *smedwi-1*. Anterior is up, and the eye and trail are in an anterior blastema at day 6 of regeneration following decapitation. Fluorescent images are FISH. Note that SMEDWI-1 protein is a marker for neoblasts and their immediate descendants, whereas *smedwi-1* mRNA labels only neoblasts. Scale bars, 50 µm.
(PDF)

Figure S12 Higher magnification images of RNAi phenotypes. All fluorescence is FISH. Anterior is up, fixed animals are on day 7 of regeneration. Arrowheads indicate double-positive cells. Scale bars, 50 µm.
(PDF)

Figure S13 Detection of presence of pigment cups by DIC. Specimens are the same as in Figure 6 and Figure S12. Anterior is up, and arrows indicate presence of pigment cup. Scale bar, 100 µm.
(PDF)

Figure S14 RNAi of *dlx* and *sp6-9* during regeneration leads to smaller pigment cups. Animals were injected with dsRNA on the indicated day of regeneration and pigment cups and terminal trail cells were visualized by *tyrosinase* expression at day 7 of regeneration. Graph shows average area of *tyrosinase^+^* pigment cup primordium in µm^2^. Error bars are s.e.m; n>12 eyes for each category; significance by two-tailed t-test is shown relative to second bar control, ***P<.001.
(PDF)

Video S1 Three-dimensional reconstruction of a pigmented optic cup from *S. mediterranea*. Pigment cup on day 6 of regeneration labeled for *tyrosinase* expression by FISH. Hollow center of the cup is visible. Individual cells, isolated from the cup, are progenitors at late stages of differentiation.
(MOV)
