# Supplementary figures and images for: dlx and sp6-9 Control Optic Cup Regeneration in a Prototypic Eye
Source: PLoS Genet. 2011 Aug 11;7(8):e1002226. doi: 10.1371/journal.pgen.1002226 (PMC3154955; doi:10.1371/journal.pgen.1002226)

Supporting Information Figure S1

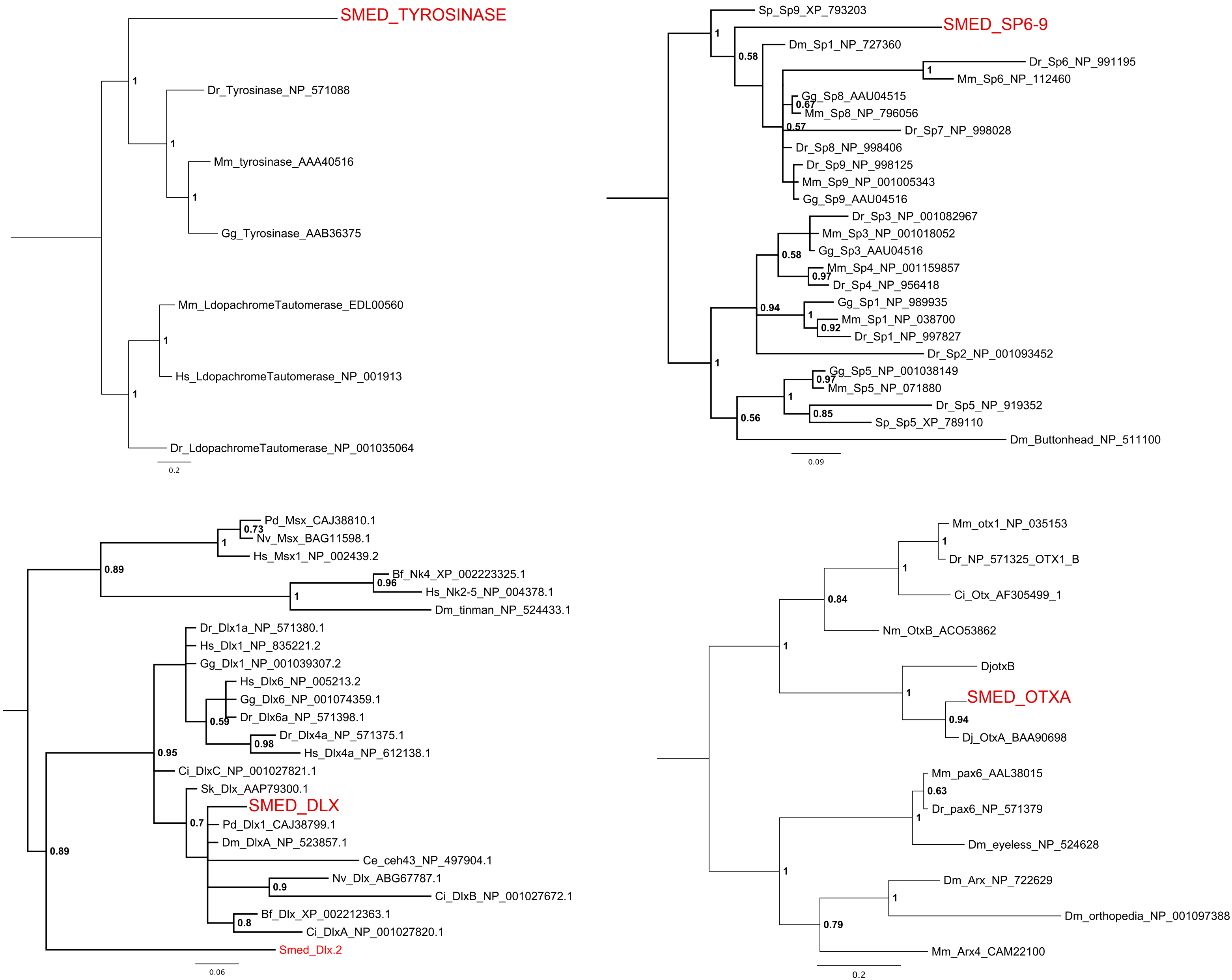

Supplement: Figure S1 — Orthology of S. mediterranea genes. Posterior probabilities are shown on branches. See Materials and Methods for details. Dm, Drosophila melanogaster; Dr, Danio rerio; Hs, Homo sapiens; Gg, Gallus gallus; Gt, Girardia tigrina; Dj, Dugesia japonica; Nv, Nematostella vectensis; Sp, Strongylocentrotus purpuratus; Pd, Platynereis dumerilii; Ci, Ciona intestinalis; Sk, Saccoglossus kowalevskii; Bf, Branchiostoma floridae; Ce, Caenorhabditis elegans; Cb, Caenorhabditis briggsae. (PDF) [file pgen.1002226.s001.pdf]

Supporting Information Figure S2

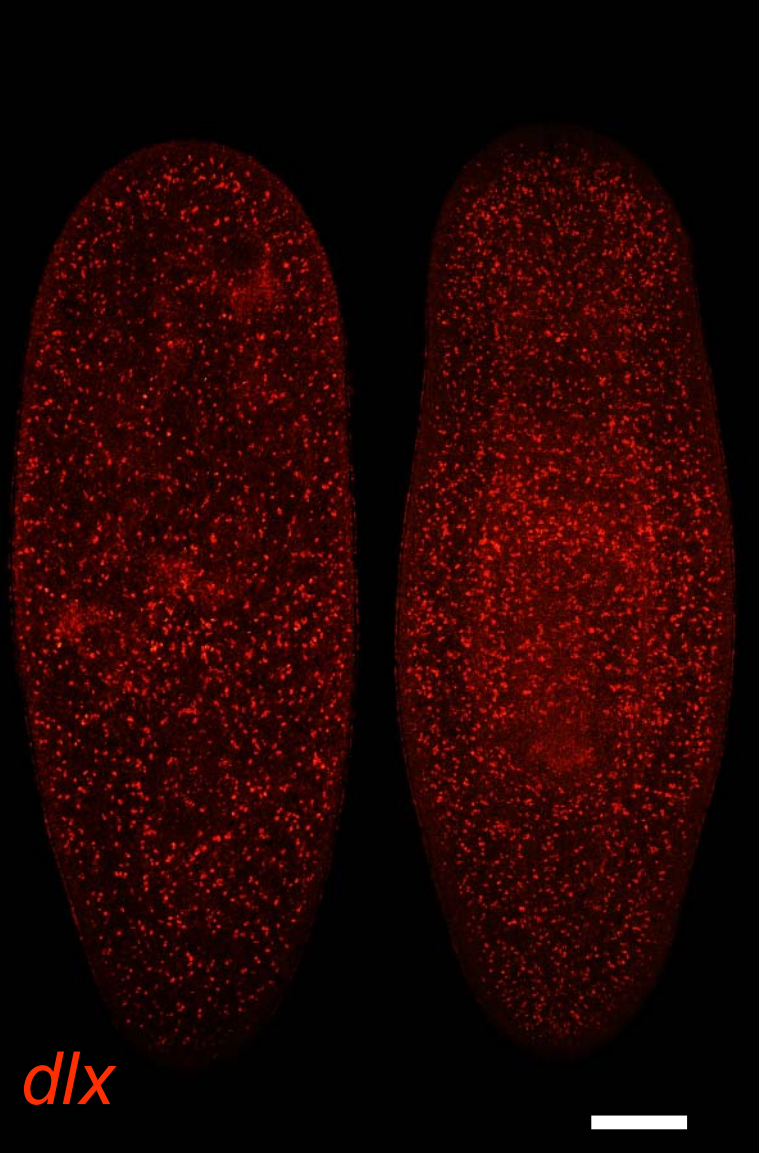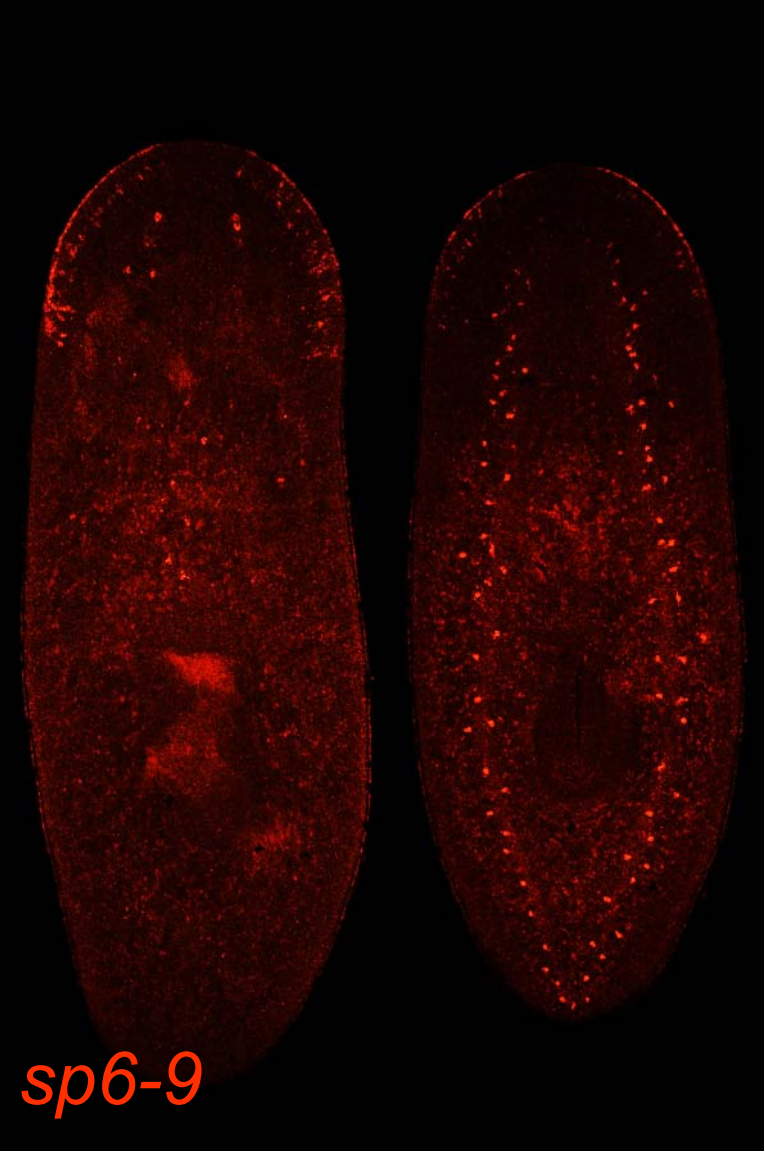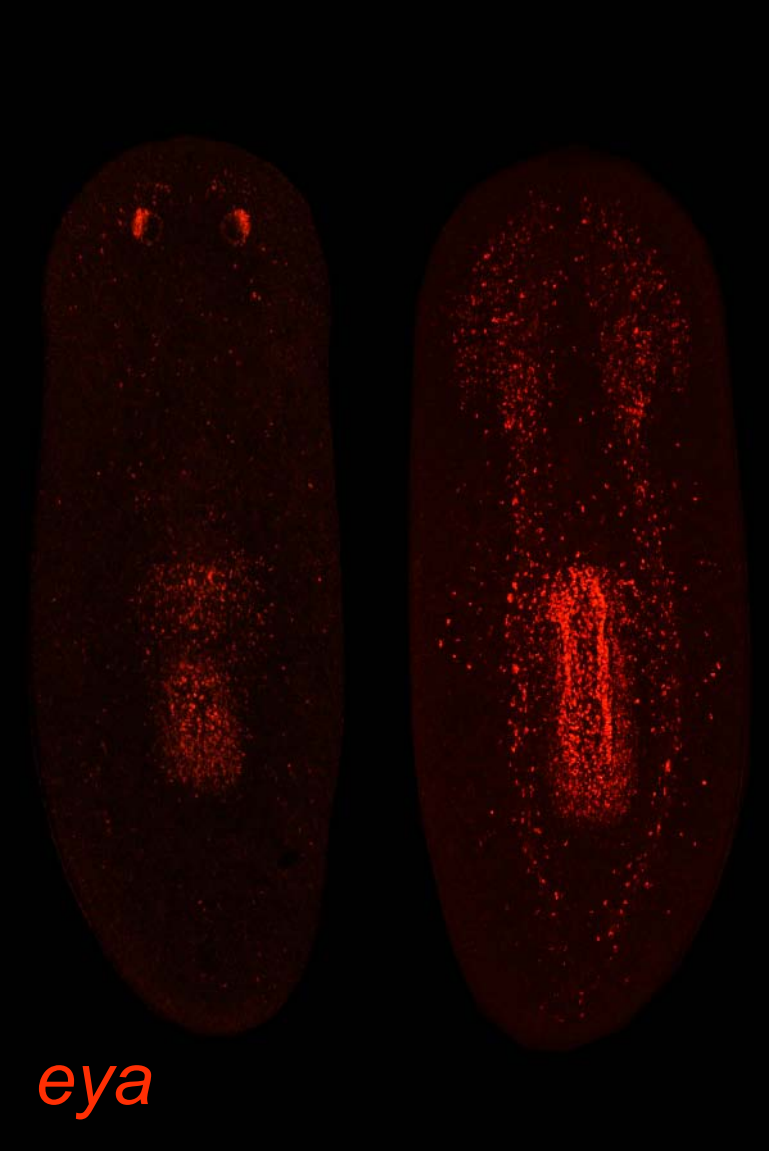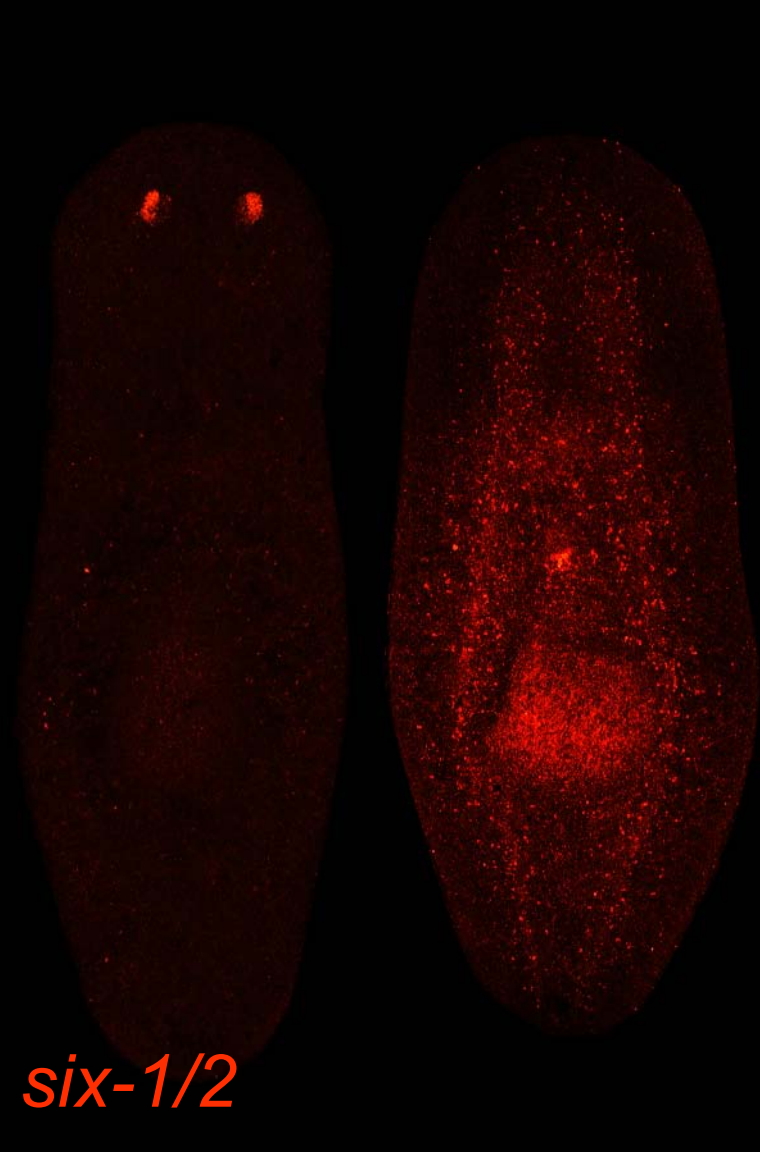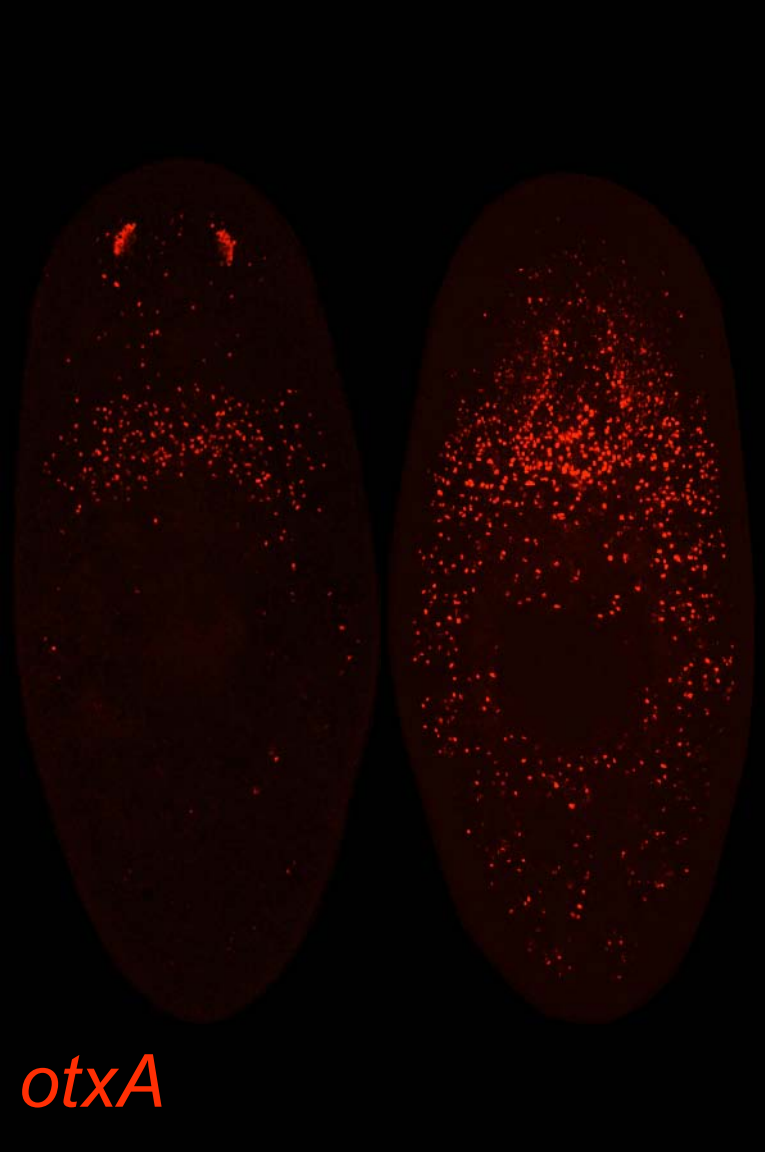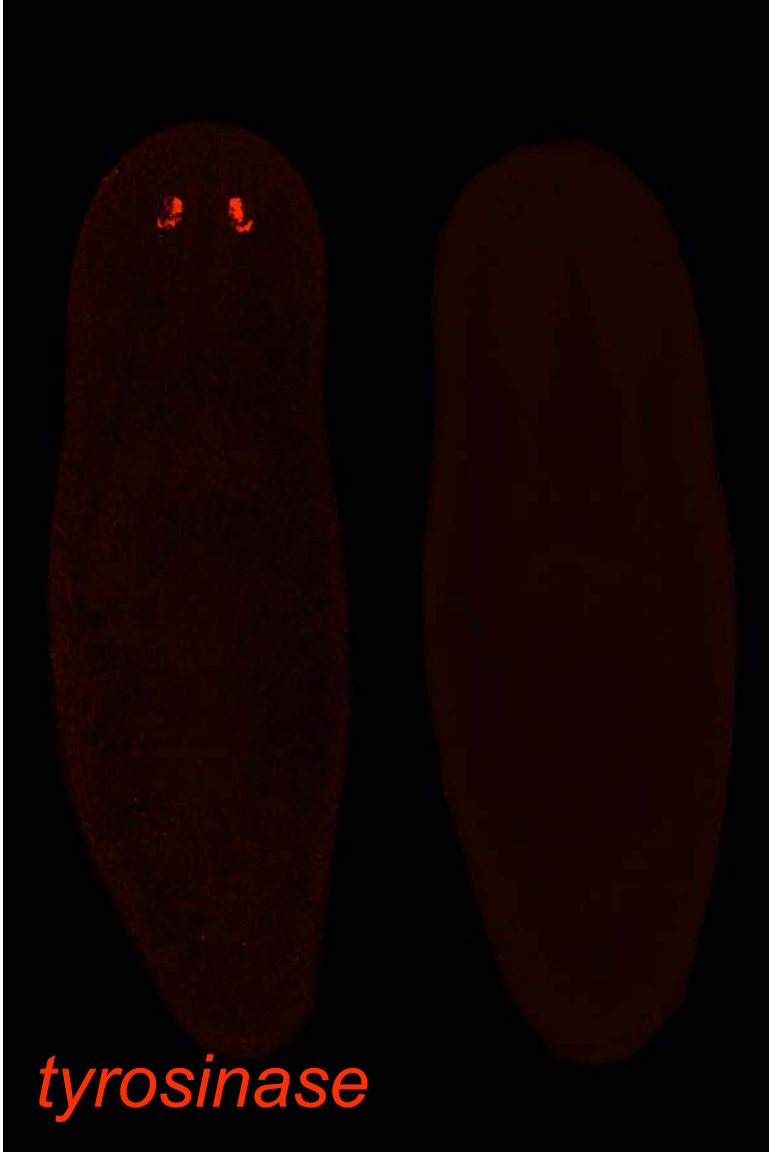

Supplement: Figure S2 — Expression of eye genes in intact animals. FISH showing expression of indicated gene in whole-mount intact animals. Expression of some pigment cup-expressed genes in intact animals is partly obscured by unbleachable eye pigment. Dorsal is shown in left panel, ventral on right for each gene. Scale bars, 200 µm. (PDF) [file pgen.1002226.s002.pdf]

Supporting Information Figure S3

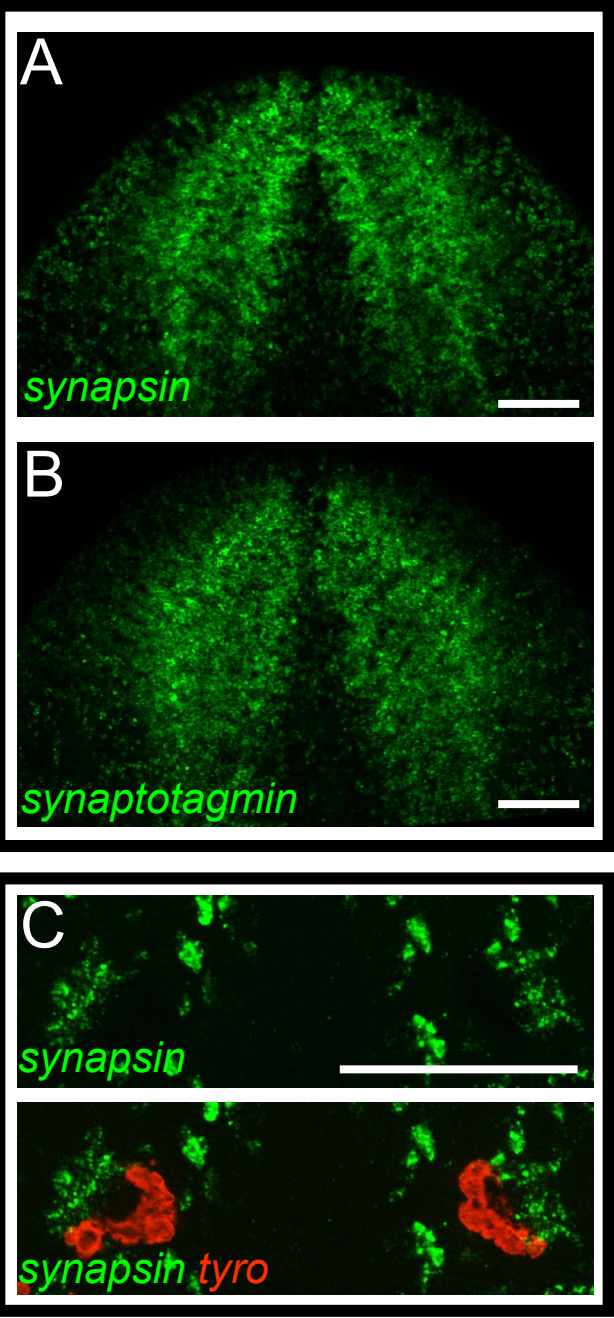

Supplement: Figure S3 — synapsin and synaptotagmin are pan-neuronally expressed and synapsin does not label the pigment cup. All fluorescence is FISH. Ventral view of 6 day regenerating animals showing pan-neuronal expression of (A) synapsin (B) synaptotagmin orthologs in the brain and elsewhere. As for synaptotagmin (Figure 1D), synapsin signal (C) is not detected in the optic cup. Scale bars, 100 µm. (PDF) [file pgen.1002226.s003.pdf]

Supporting Information Figure S4

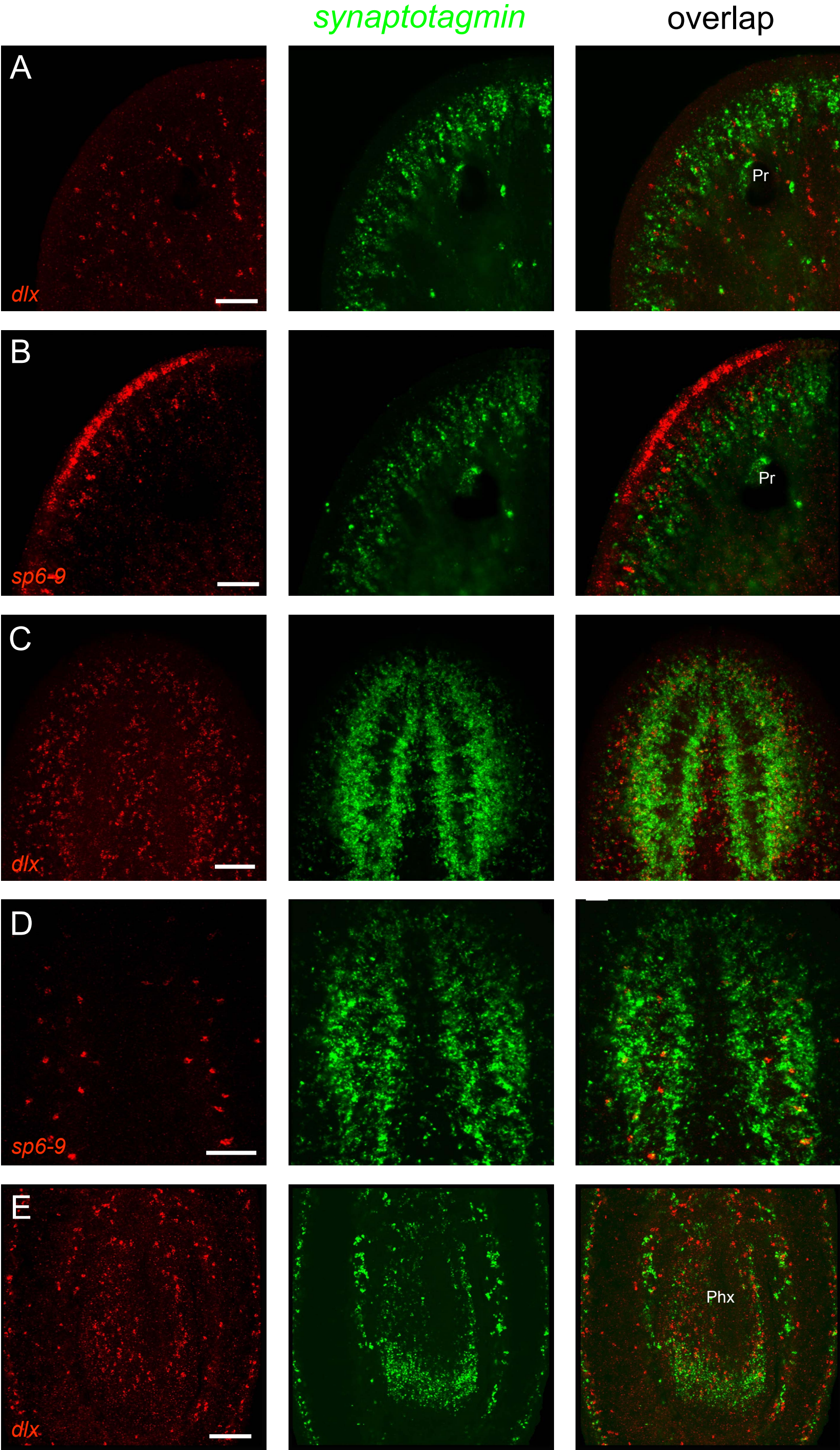

Supplement: Figure S4 — Expression domains of dlx and sp6-9 in relation to neurons of the head rim and brain. All fluorescence is FISH, animals are intact (non-regenerating), and anterior pole is facing up. (A) and (B) are dorsal views of the anterior left side of the animal, (C) and (D) are ventral views of the anterior of the animal. (A) dlx is expressed in diffuse cells throughout the dorsal anterior, some of which express neuronal markers. (B) sp6-9 is prominently expressed in the head rim epidermis, as well as sparse underlying cells. These cells abut neurons but largely do not express synaptotagmin. (C) dlx is expressed in many neurons within the primary lobes of the brain as well as more lateral regions. (D) sp6-9 is expressed in a small subset of cells located in the ventral brain, most of which express synaptotagmin. (E) dlx is also expressed in cells of the pharynx and pharynx cavity at the midbody of the animal. Pr, photoreceptors; Phx: pharynx. Scale bars, 50 µm. (PDF) [file pgen.1002226.s004.pdf]

Supporting Information Figure S5

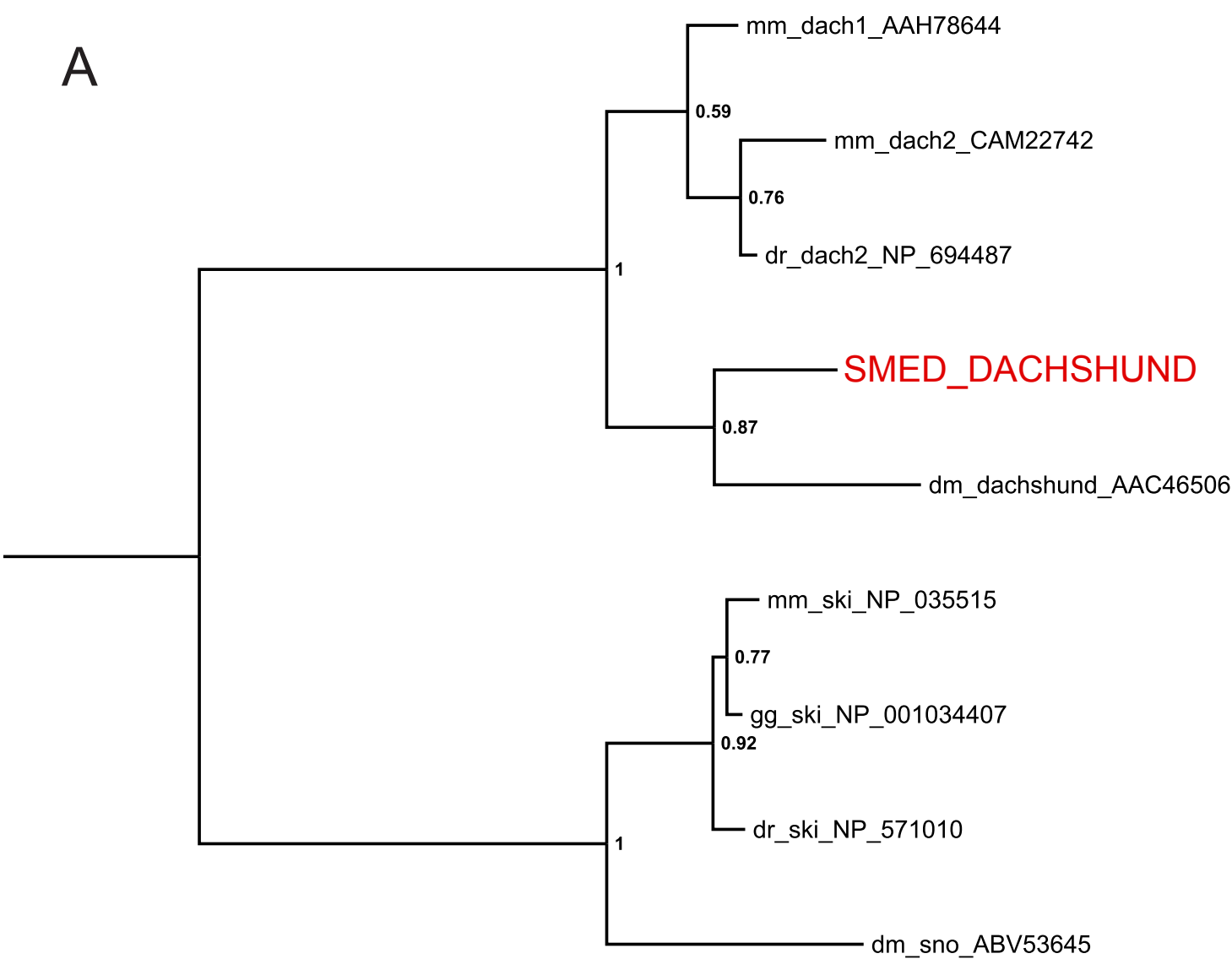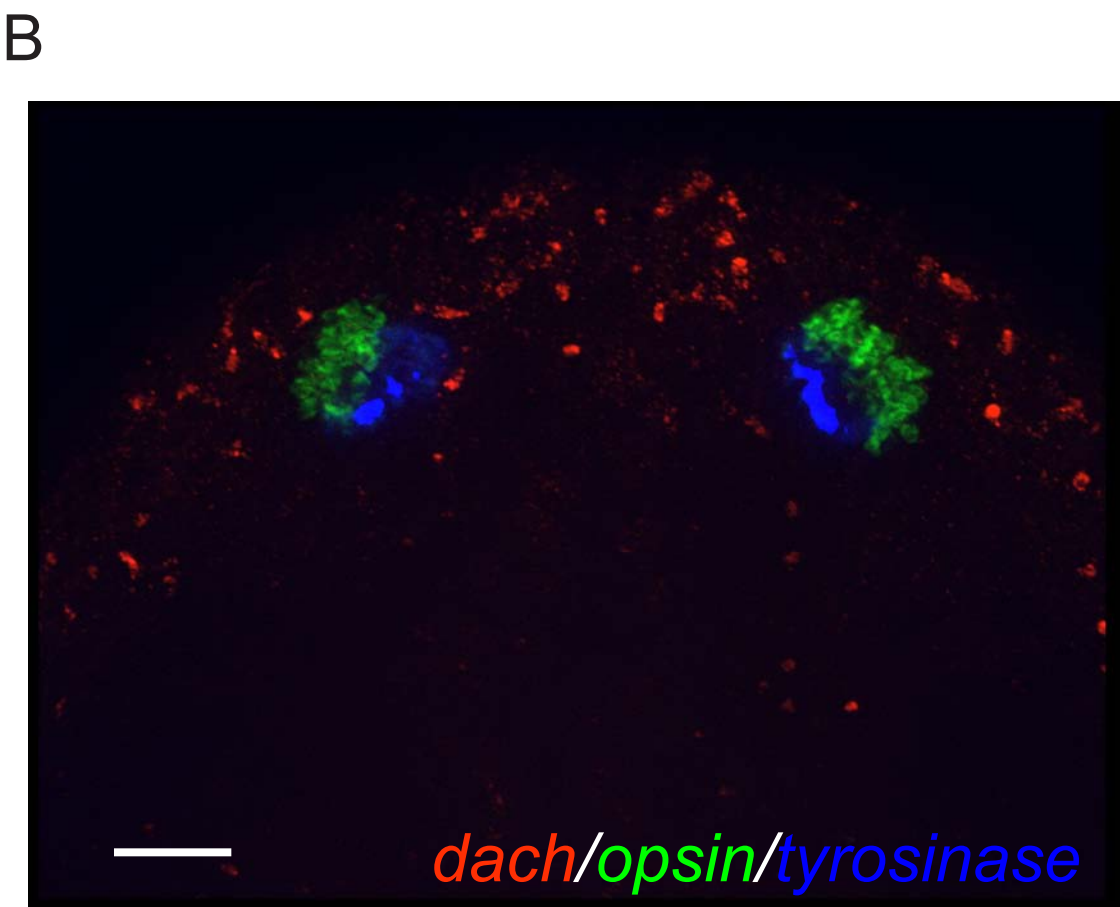

Supplement: Figure S5 — dachshund is not detectably expressed in the regenerating eye and does not have an obvious eye phenotype following dsRNA injection. (A) Orthology of Smed-dachshund. Posterior probabilities are shown on branches, see Materials and Methods for details. dm, Drosophila melanogaster; dr, Danio rerio; gg, Gallus gallus; mm, mus musculus. (B) Expression analysis with FISH does not indicate expression of Smed-dachshund in the photoreceptor neurons or the optic cup in 6 day regenerating heads. opsin (green) and tyrosinase (blue) expression are used to label photoreceptor neurons and optic cup, respectively. Scale bar, 50 µm. (PDF) [file pgen.1002226.s005.pdf]

Supporting Information Figure S6

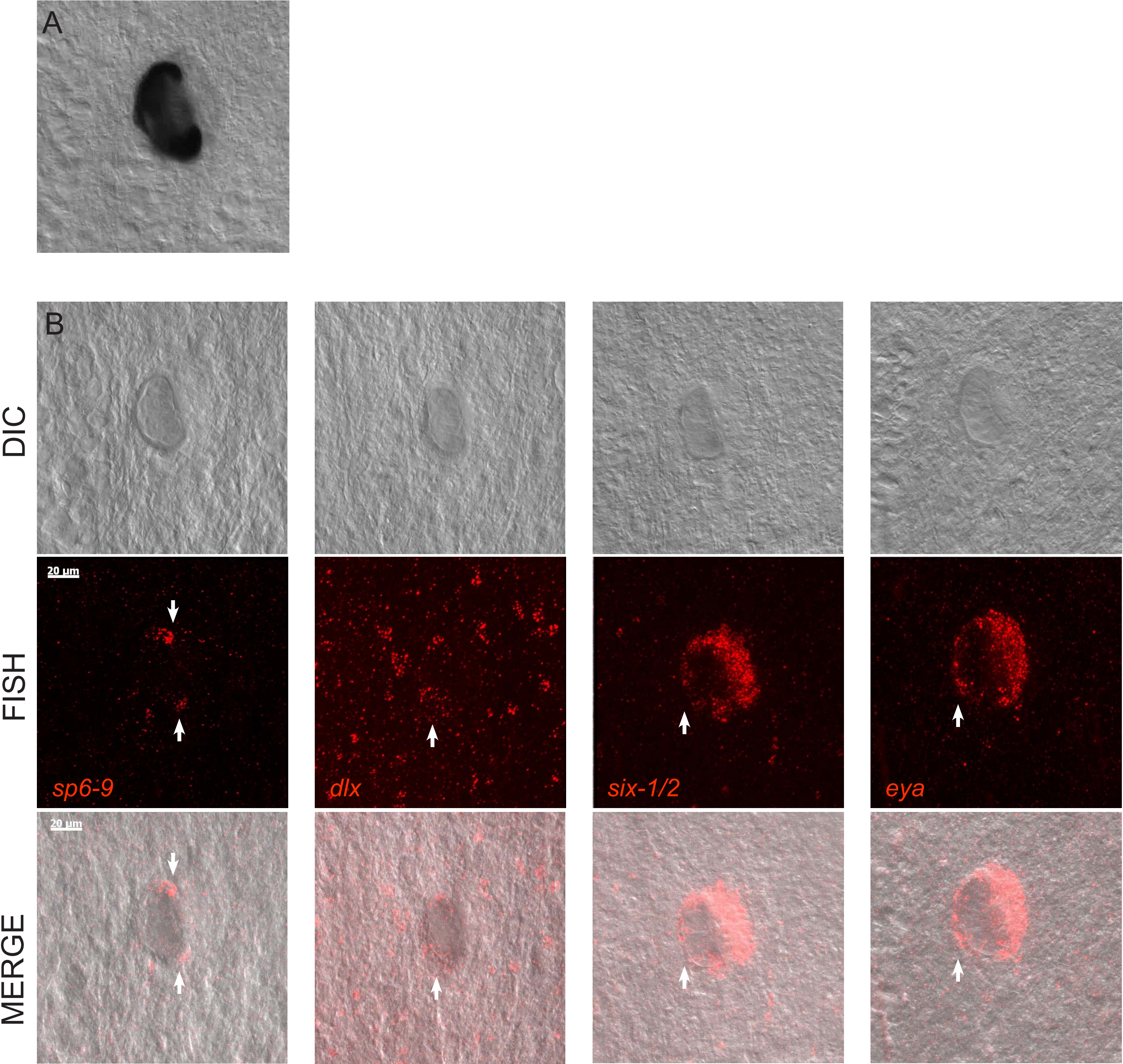

Supplement: Figure S6 — Expression of transcription factors in de-pigmented optic cups of intact animals. RNAi of tyrosinase was used to reduce melanin pigment in the optic cup. (A) DIC image of an optic cup in an animal not treated with tyrosinase RNAi. (B) Expression of selected transcription factors in intact eyes of tyrosinase RNAi animals (FISH). The crescent-shaped structure in the DIC image is the optic cup. Arrows indicate regions of the optic cup with expression of the gene labeled in the panel. Scale bar, 20 µm. (PDF) [file pgen.1002226.s006.pdf]

Supporting Information Figure S7

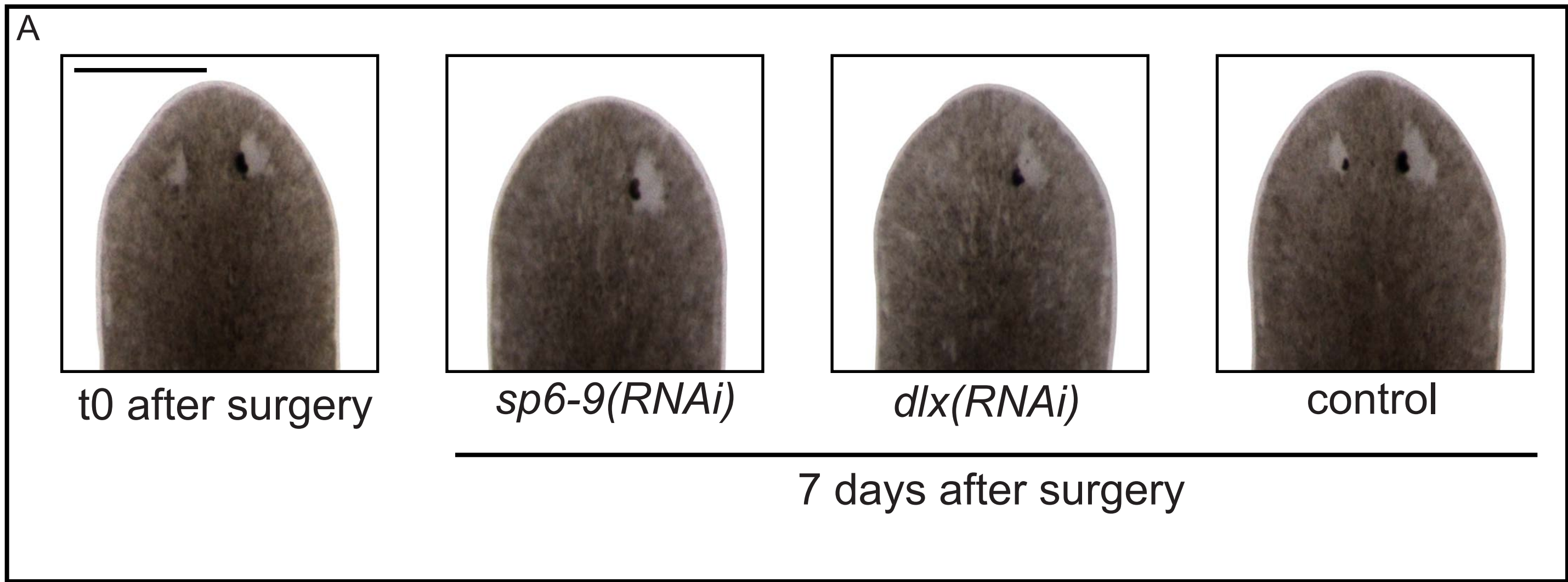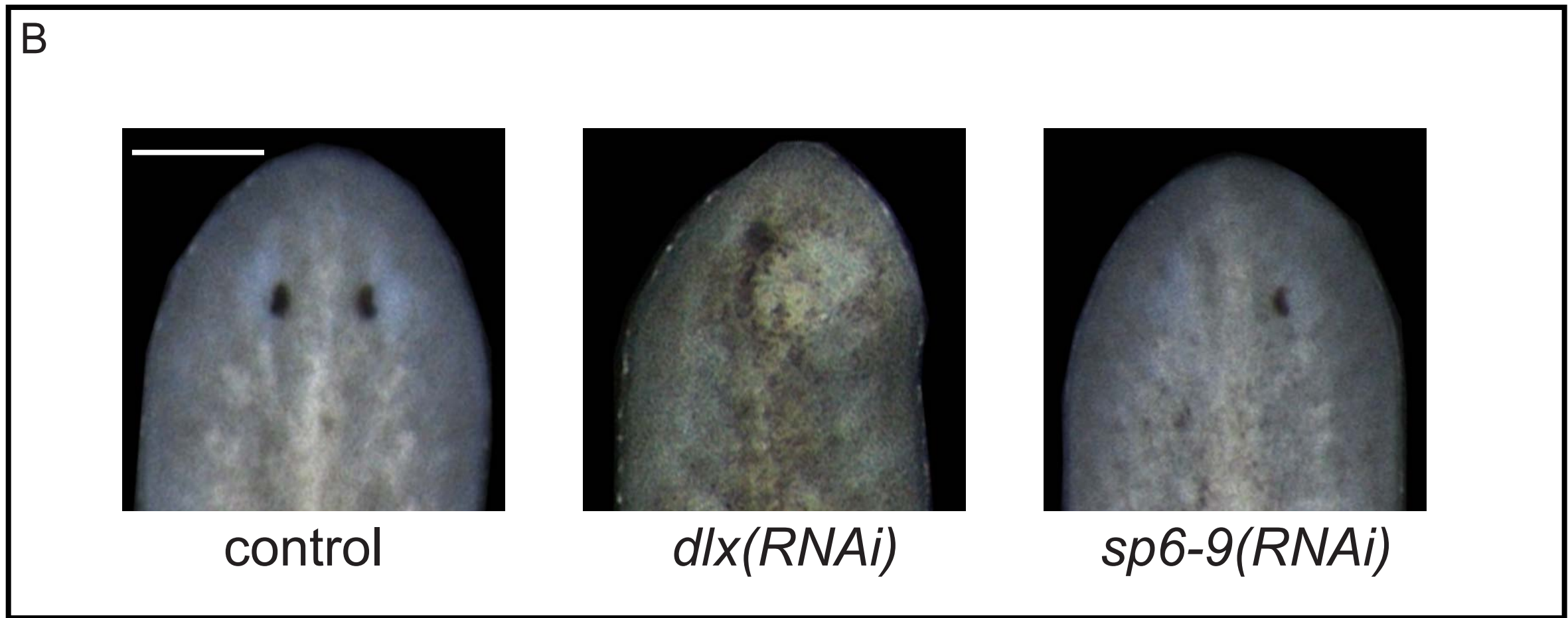

Supplement: Figure S7 — sp6-9 and dlx are required for regeneration of the optic cup after excision and for homeostatic maintenance of the optic cup. (A) The optic cup was surgically removed after 3 RNAi feedings, at which point animals in all RNAi conditions appeared similar. 7 days after surgery, only control animals showed signs of optic cup regeneration. Regeneration of the optic cup was apparent in n = 10/10 control RNAi animals, n = 0/10 dlx(RNAi) animals and n = 0/10 sp6-9(RNAi) animals. (B) RNAi of dlx in uninjured animals resulted in worm lysis within 3 weeks. n = 5/9 worms surviving at three weeks of homeostasis had lesions in the area of the eye. RNAi of sp6-9 in uninjured animals resulted in pigment cups that were reduced in size and pigmentation in n = 20/20 animals after 7 weeks. 4/20 of these animals lost pigment cups completely. Scale bars, 500 µm (A), 200 µm (B). (PDF) [file pgen.1002226.s007.pdf]

# Supporting Information Figure S8

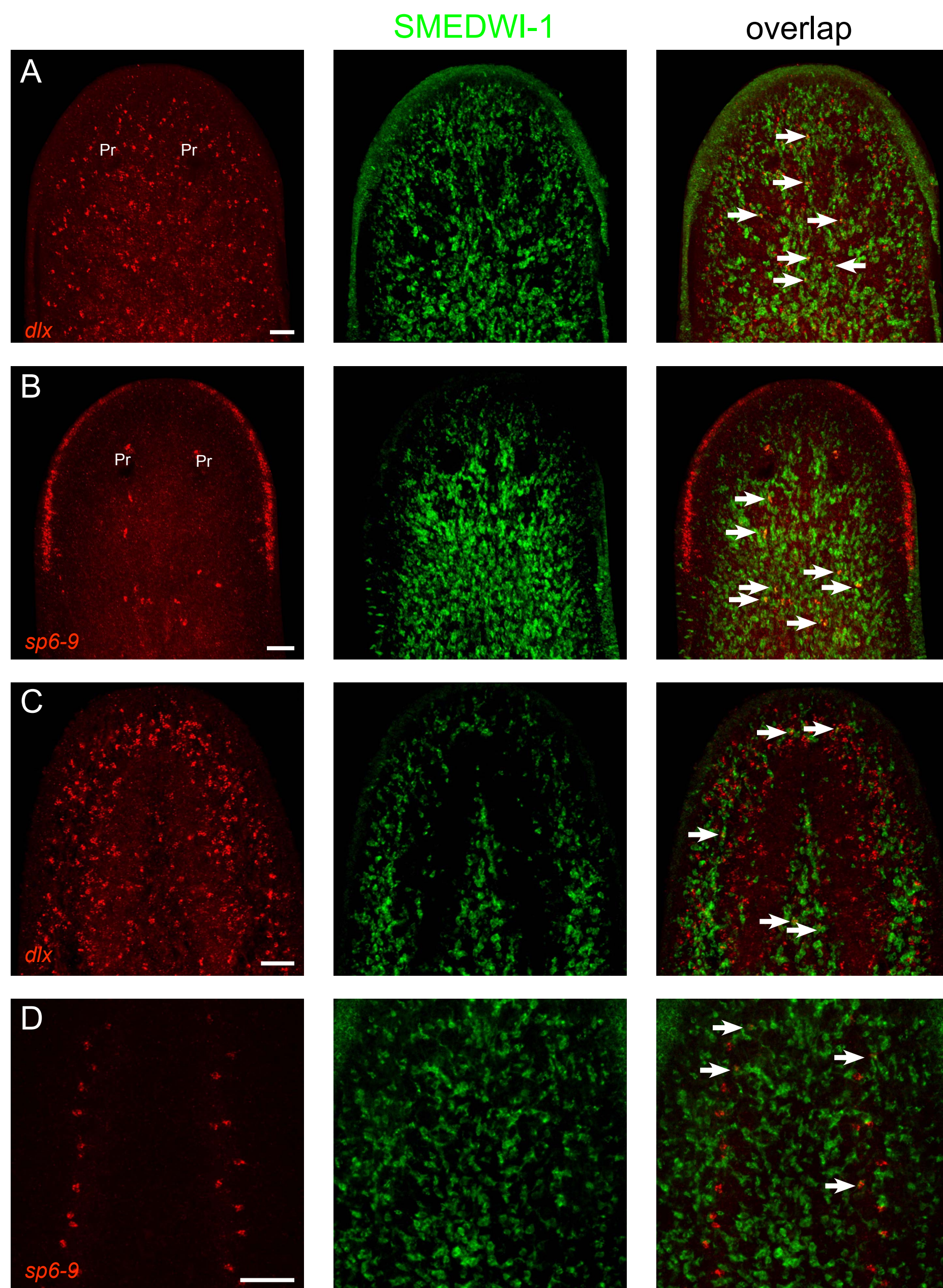

Supplement: Figure S8 — Expression of dlx and sp6-9 in neoblasts or immediate neoblast descendants in intact animals. All fluorescence is FISH, animals are intact (non-regenerating). (A) and (B) are dorsal views, (C) and (D) are ventral views. (A) dlx-expressing cells on the dorsal side of the animal that also express SMEDWI-1 protein can be found both posterior and anterior to the photoreceptors. (B) A small population of sp6-9-expressing cells in the pre-pharyngeal region posterior to the photoreceptors also expresses SMEDWI-1 protein. These may represent optic cup progenitors that function during homeostasis. (C) Some dlx-expressing cells at the periphery of the brain lobes are positive for SMEDWI-1. (D) Most sp6-9 positive neurons (located in a ventral region of the brain) are fully differentiated, but some cells in the anterior of this domain express SMEDWI-1. Scale bars, 50 µm. (PDF) [file pgen.1002226.s008.pdf]

Supporting Information Figure S9

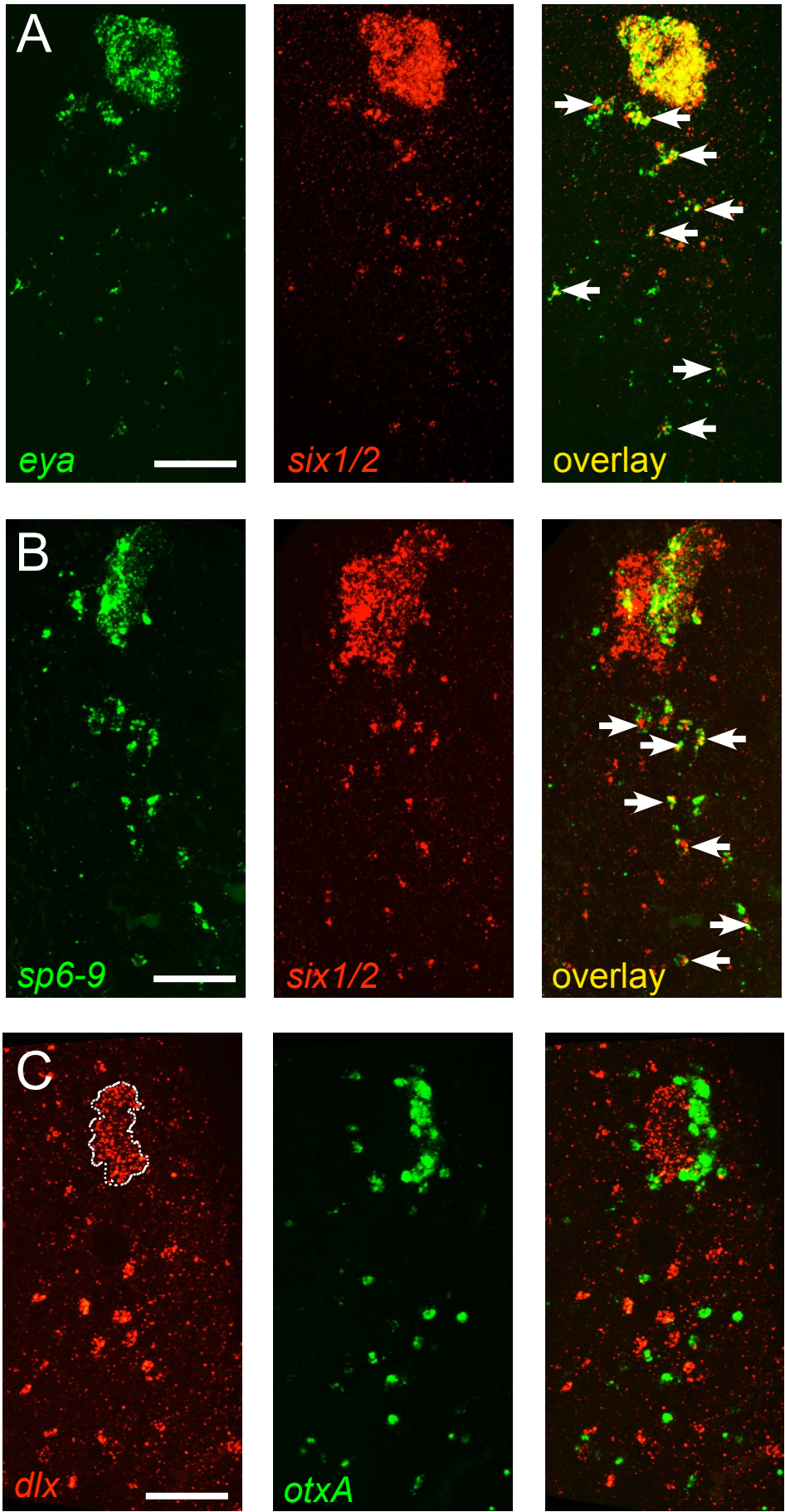

Supplement: Figure S9 — Additional image data for Figure 3. Anterior is up in all images, and all eyes and trails are in anterior blastemas at day 6 of regeneration following decapitation. Fluorescent images are FISH. (A) six1/2 expression and eya expression overlap fully in the eye aggregate and in the trail. (B) six1/2 expression and sp6-9 expression overlap partly in the eye aggregate and partly in the trail. (C) dlx-expressing cells do not detectably express otxA. Arrowheads indicate double-positive cells. Scale bars, 100 µm. (PDF) [file pgen.1002226.s009.pdf]

Supporting Information Figure S10

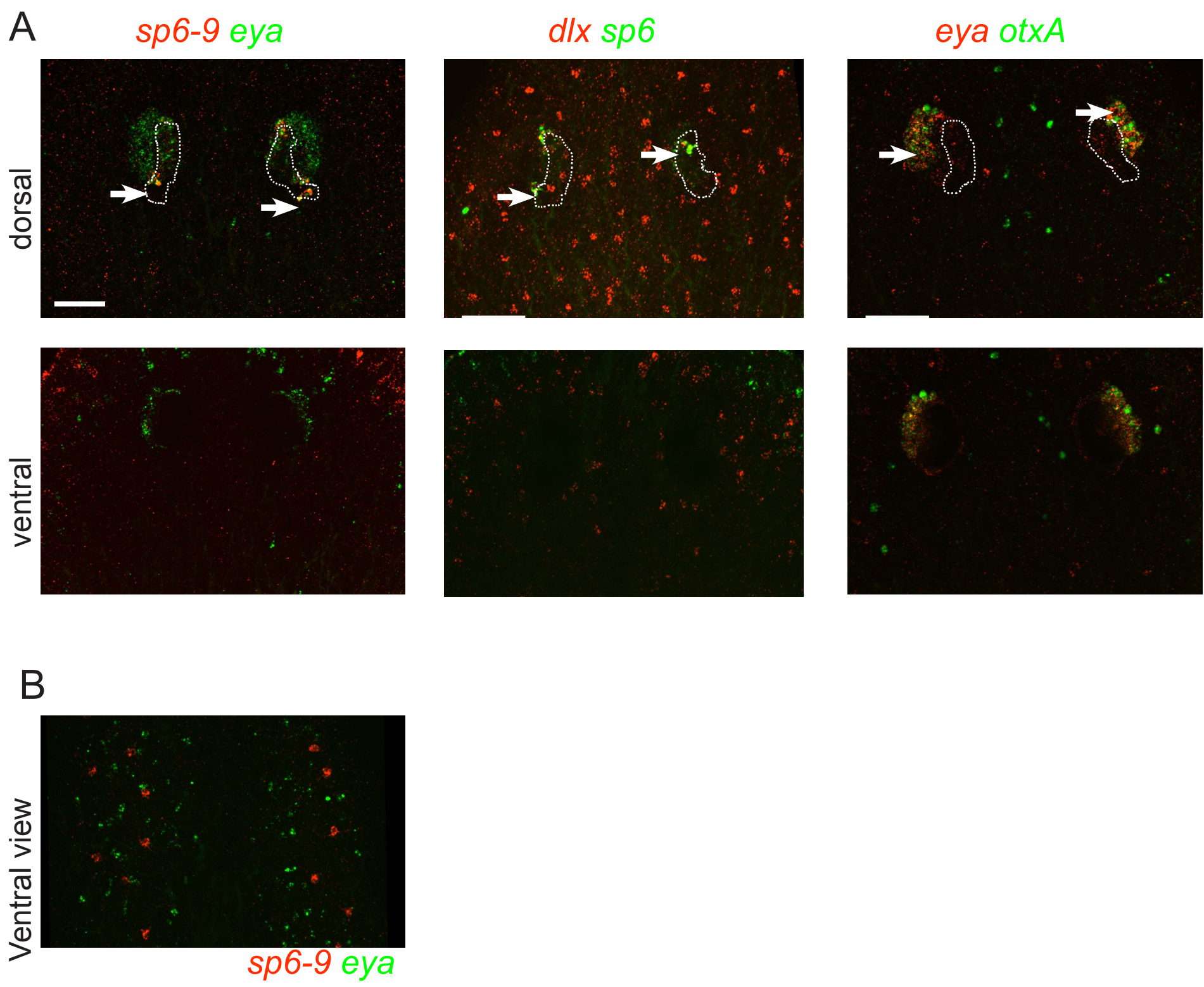

Supplement: Figure S10 — Overlapping expression of transcription factor combinations is not found outside of the eye region. (A) FISH showing expression of indicated genes in whole mount intact animals. Both rows show overlayed optical sections imaged from the dorsal surface. The second row is imaged at a more ventral level than the first. Arrows indicate regions with double positive cells. In intact animals, sp6-9, dlx, and eya expression is difficult to detect in intact pigment cups, but is apparent in new cells that are incorporating as part of homeostatic maintenance. (B) Overlayed optical sections taken from ventral surface of the animal showing that sp6-9+ nerve cord cells do not detectably express eya, unlike optic cup cells. Scale bar, 50 µm. (PDF) [file pgen.1002226.s010.pdf]

Supporting Information Figure S11

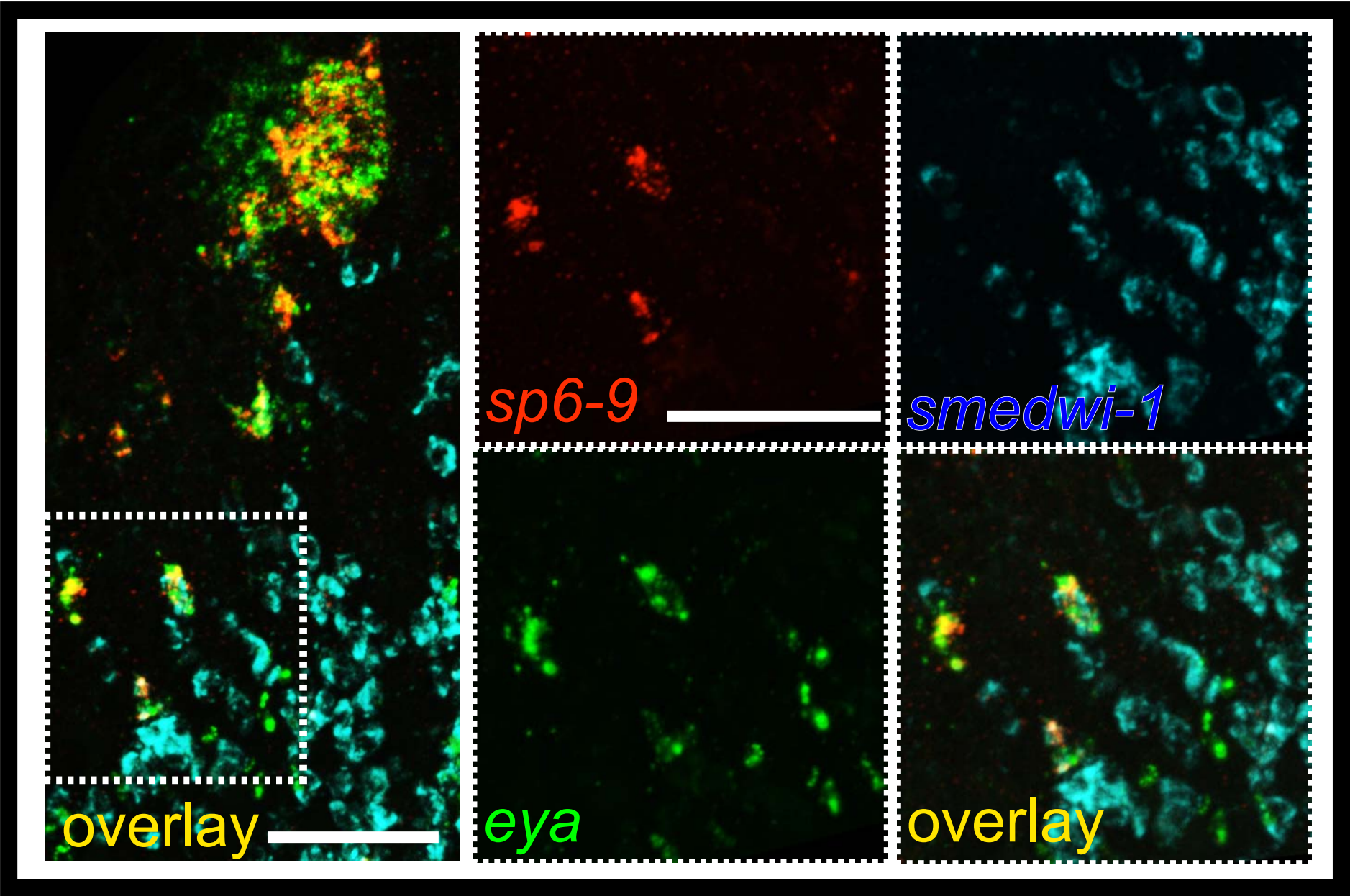

Supplement: Figure S11 — Some sp6-9+/eya+ trail cells also express smedwi-1. Anterior is up, and the eye and trail are in an anterior blastema at day 6 of regeneration following decapitation. Fluorescent images are FISH. Note that SMEDWI-1 protein is a marker for neoblasts and their immediate descendants, whereas smedwi-1 mRNA labels only neoblasts. Scale bars, 50 µm. (PDF) [file pgen.1002226.s011.pdf]

Supporting Information Figure S12

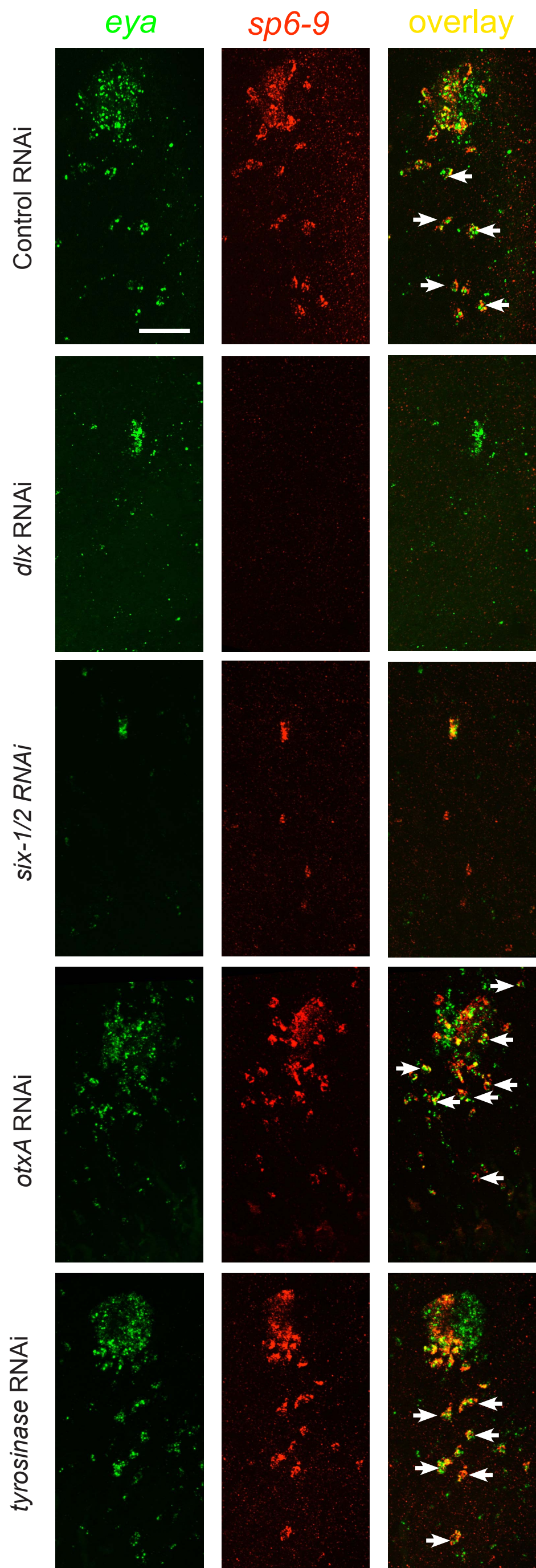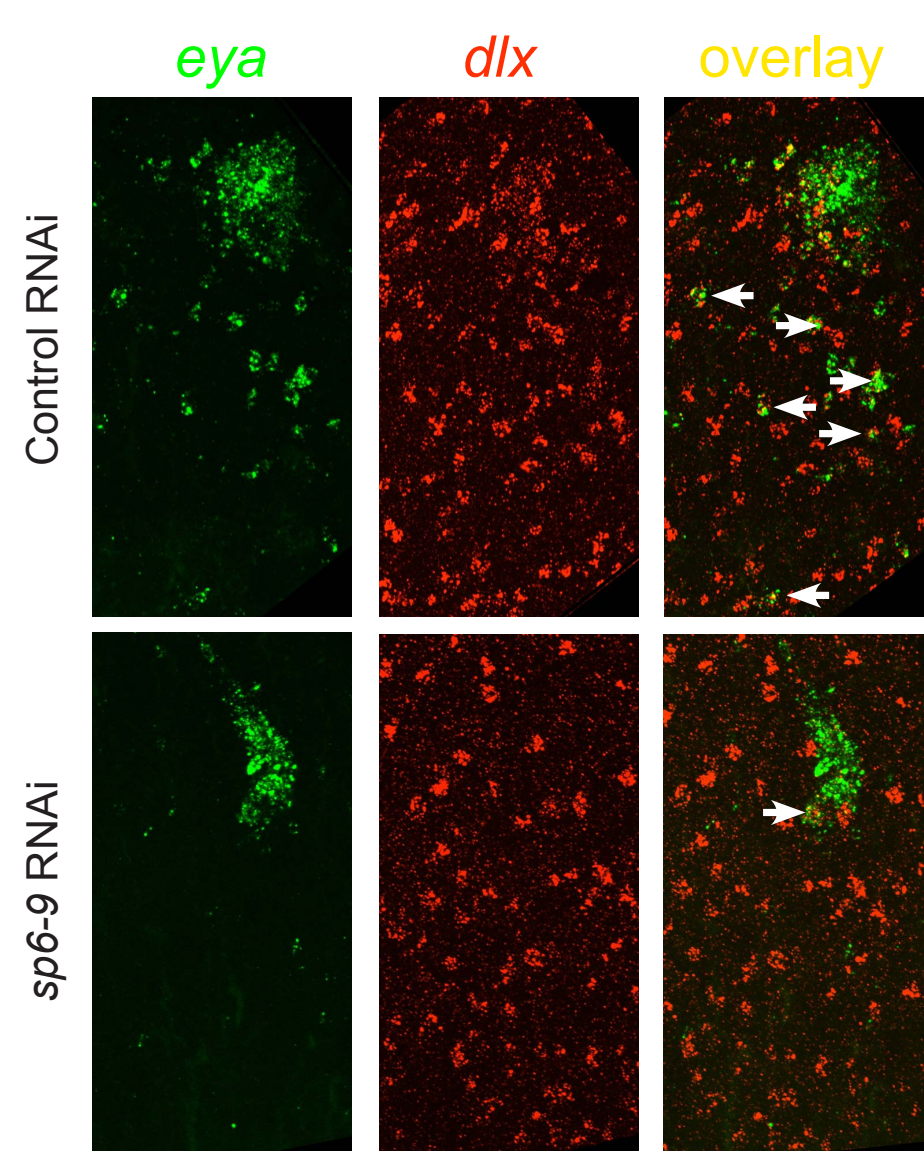

Supplement: Figure S12 — Higher magnification images of RNAi phenotypes. All fluorescence is FISH. Anterior is up, fixed animals are on day 7 of regeneration. Arrowheads indicate double-positive cells. Scale bars, 50 µm. (PDF) [file pgen.1002226.s012.pdf]

Supporting Information Figure S14

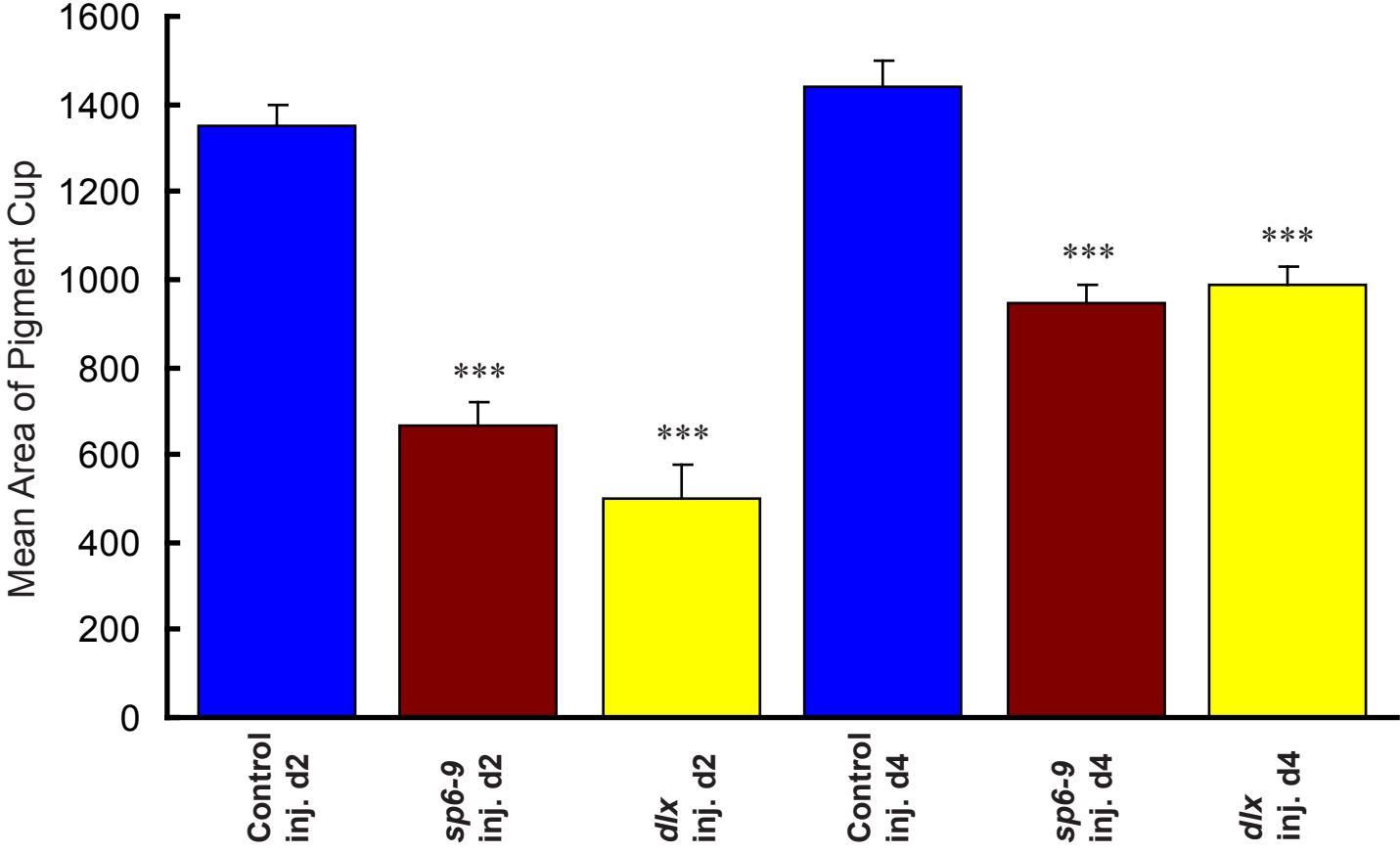

Supplement: Figure S14 — RNAi of dlx and sp6-9 during regeneration leads to smaller pigment cups. Animals were injected with dsRNA on the indicated day of regeneration and pigment cups and terminal trail cells were visualized by tyrosinase expression at day 7 of regeneration. Graph shows average area of tyrosinase+ pigment cup primordium in µm2. Error bars are s.e.m; n>12 eyes for each category; significance by two-tailed t-test is shown relative to second bar control, ***P<.001. (PDF) [file pgen.1002226.s014.pdf]
