# Supplementary material for: dlx and sp6-9 Control Optic Cup Regeneration in a Prototypic Eye
Source: PLoS Genet. 2011 Aug 11;7(8):e1002226. doi: 10.1371/journal.pgen.1002226 (PMC3154955; doi:10.1371/journal.pgen.1002226)

Supporting Information Figure S13

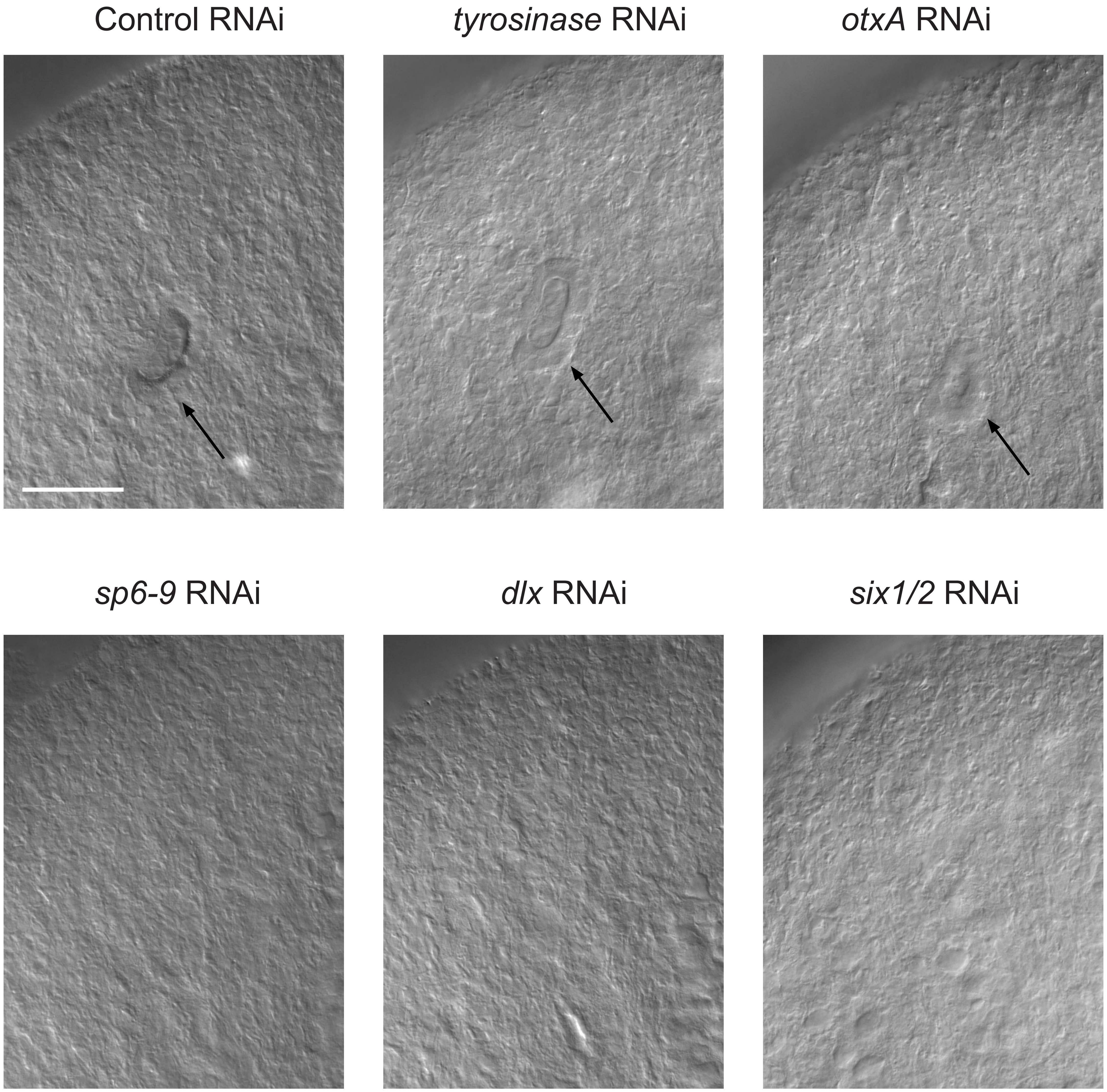

Supplement: Figure S13 — Detection of presence of pigment cups by DIC. Specimens are the same as in Figure 6 and Figure S12. Anterior is up, and arrows indicate presence of pigment cup. Scale bar, 100 µm. (PDF) [file pgen.1002226.s013.pdf]
